# Supplementary material for: ZFHX4 is necessary for dopaminergic neuron differentiation and controls cell cycle by regulating LIN28A
Source: Stem Cell Reports. 2026 May 28;21(6):102930. doi: 10.1016/j.stemcr.2026.102930 (PMC13261933; doi:10.1016/j.stemcr.2026.102930)
Supplement: Document S1. Figures S1–S5 [file mmc1.pdf]

Stem Cell Reports, Volume *21*

Supplemental Information

ZFHX4 is necessary for dopaminergic neuron differentiation and controls cell cycle by regulating LIN28A

Elena Valceschini, Borja Gomez Ramos, Jochen Ohnmacht, Aurelien Ginolhac, Marie Catillon, Deborah Gerard, Anthoula Gaigneaux, Dimitrios Kyriakis, Kamil Grzyb, Enrico Glaab, Anne Grünewald, Alexander Skupin, Thomas Sauter, Rejko Krüger, and Lasse Sinkkonen

Figure S1

A

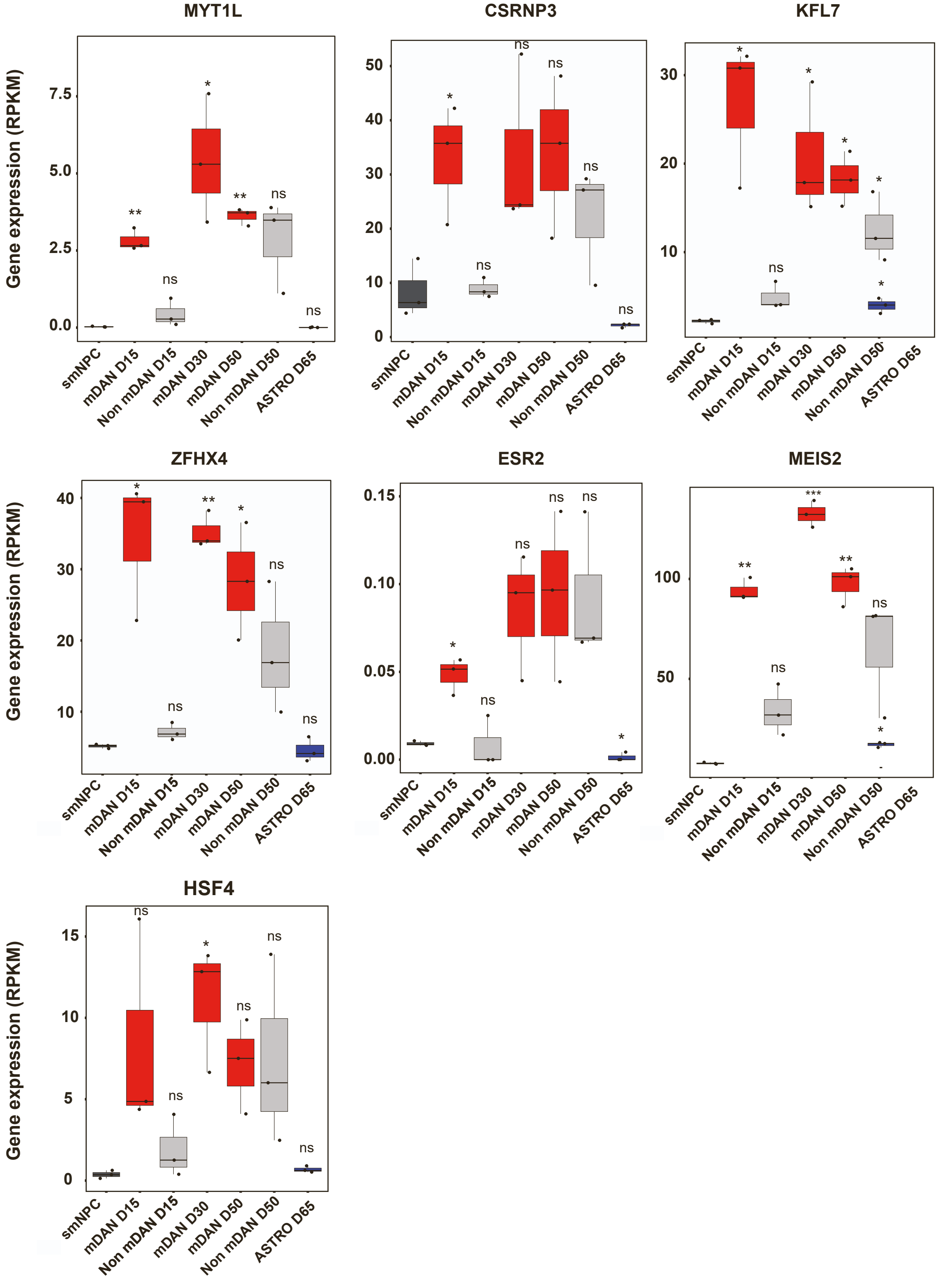

Figure S1: Expression of 7 TFs across mDAN differentiation. A) Expression dynamics of the 7 candidate TFs during mDAN differentiation of the TH-Rep1 cell line.

Figure S2

A

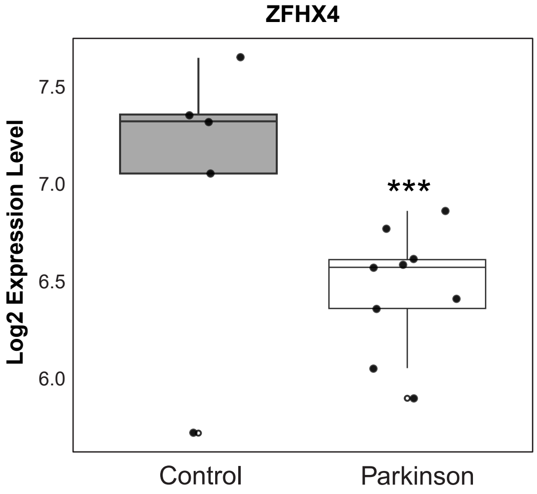

Figure S2: ZFHX4 expression is reduced in PD. A) Box plot showing ZFHX4 expression levels in lateral substantia nigra tissue from PD patients and healthy controls. Individual data points are overlaid on box plots, showing median, interquartile range, and outliers.

Figure S3

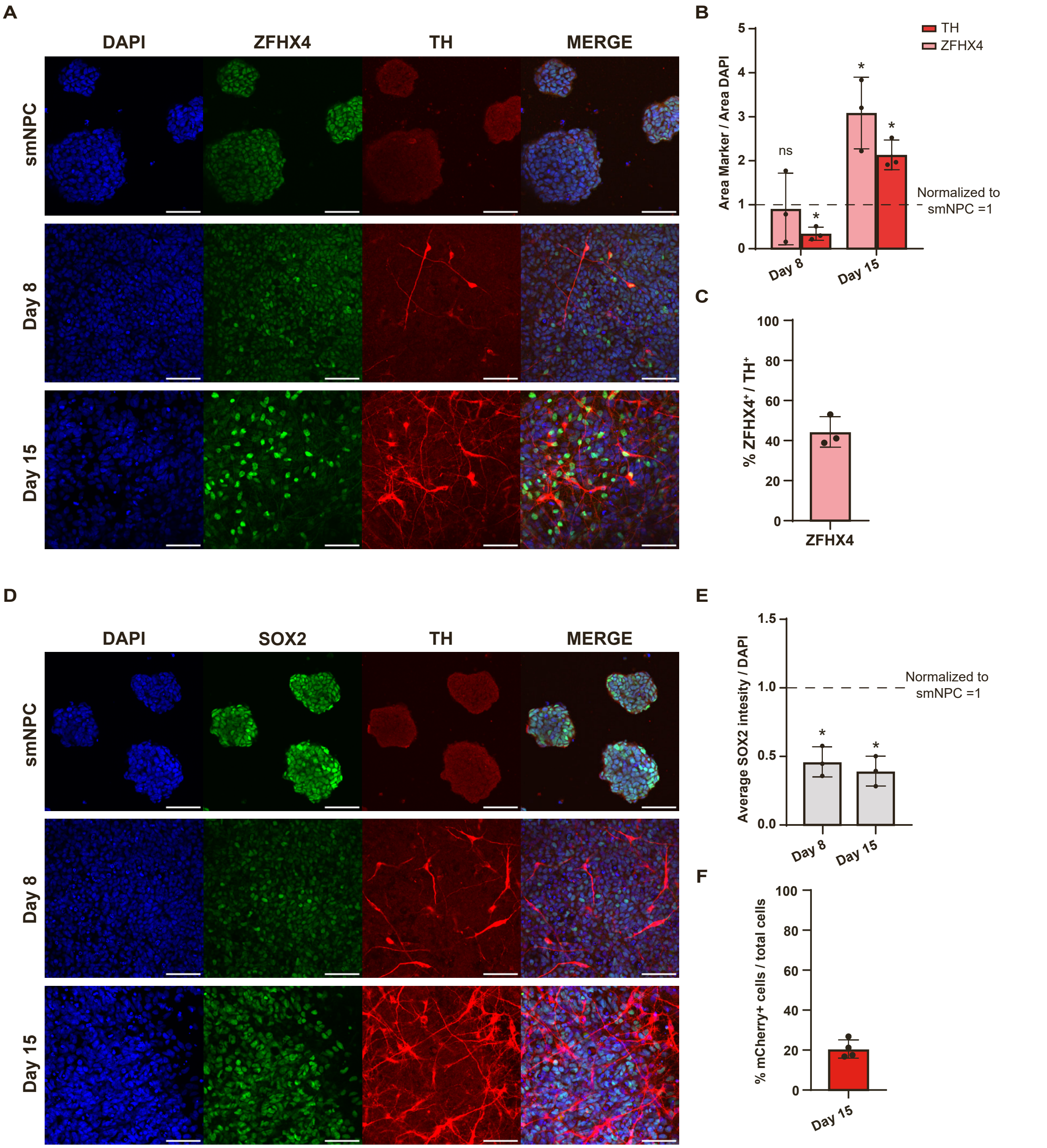

Figure S3: mDAN maturation requires the induction of ZFHX4. A) Representative images of the nuclear marker DAPI, ZFHX4, TH and the merged signal, in smNPC, day 8 of differentiation and day 15. B) Quantification of ZFHX4 and TH Area over Area of DAPI. Ratios were normalized to smNPC within each replicate (N = 3 independent experiments). C) Proportion of cells that are both TH positive and ZFHX4 positive in terms of intensity signal (N = 3 independent experiments). D) Representative images of the nuclear marker DAPI, SOX2, TH and the merged signal in smNPC, day 8 of differentiation and day 15. E) Quantification of SOX2 and TH Area over Area of DAPI. Ratios were normalized to smNPC per replicate (N = 3 independent experiments). F) Flow cytometry-based quantification of mCherry+ neurons at day 15 in TH-Rep1 reporter cell line compared with the unreporter control cell line (N = 4 independent experiments). Error bars (B, C, E, F), correspond to ±1 standard deviation (SD) from the mean. t-test (B, E), * = p-value < 0.05, ** = p-value < 0.01, *** = p-value < 0.001, **** = p-value < 0.0001, and ns = not significant.

Figure S4

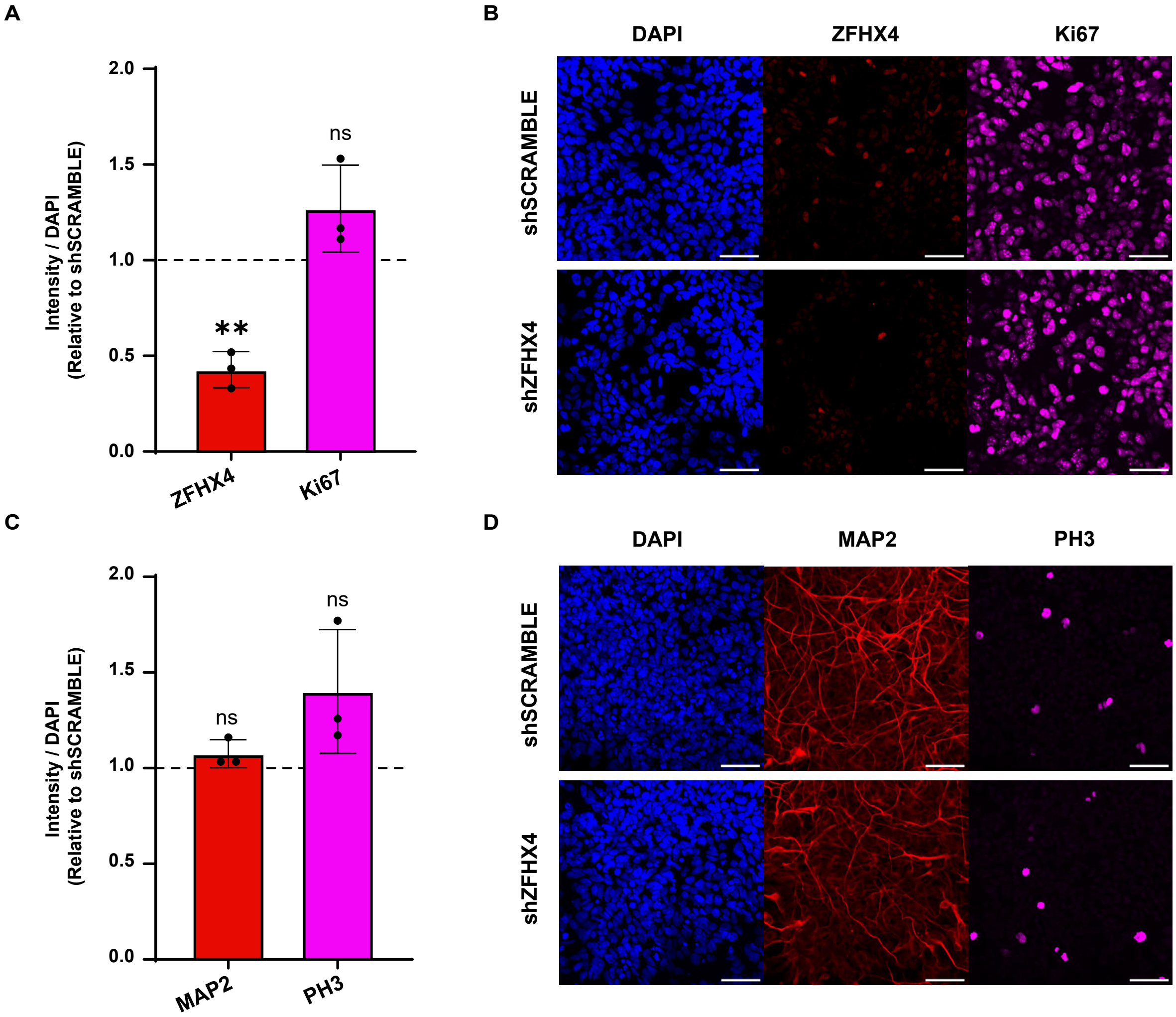

Figure S4: Proliferation markers tend to increase upon ZFHX4 depletion. A) Quantification of Ki67 or ZFHX4 positive-area normalized to DAPI-stained area. Ratios were normalized to shSCRAMBLE within each replicate. Analysis was performed on day 8 following early ZFHX4 KD (N = 3 independent experiments). (B) Representative images showing the nuclear marker DAPI, ZFHX4 and the proliferation marker Ki67. C) Quantification of MAP2 or PH3 positive-area normalized to DAPI stained area. Ratios were normalized to shSCRAMBLE within each replicate. Analysis was performed on day 8 following early ZFHX4 KD (N = 3 independent experiments). (D) Representative images of the nuclear marker DAPI, the neuronal marker MAP2 and the mitosis marker PH3. Error bars correspond to ±1 standard deviation (SD) from the mean, t-test, * = p-value < 0.05, ** = p-value < 0.01, *** = p-value < 0.001, **** = p-value < 0.0001, and ns = not significant.

Figure S5

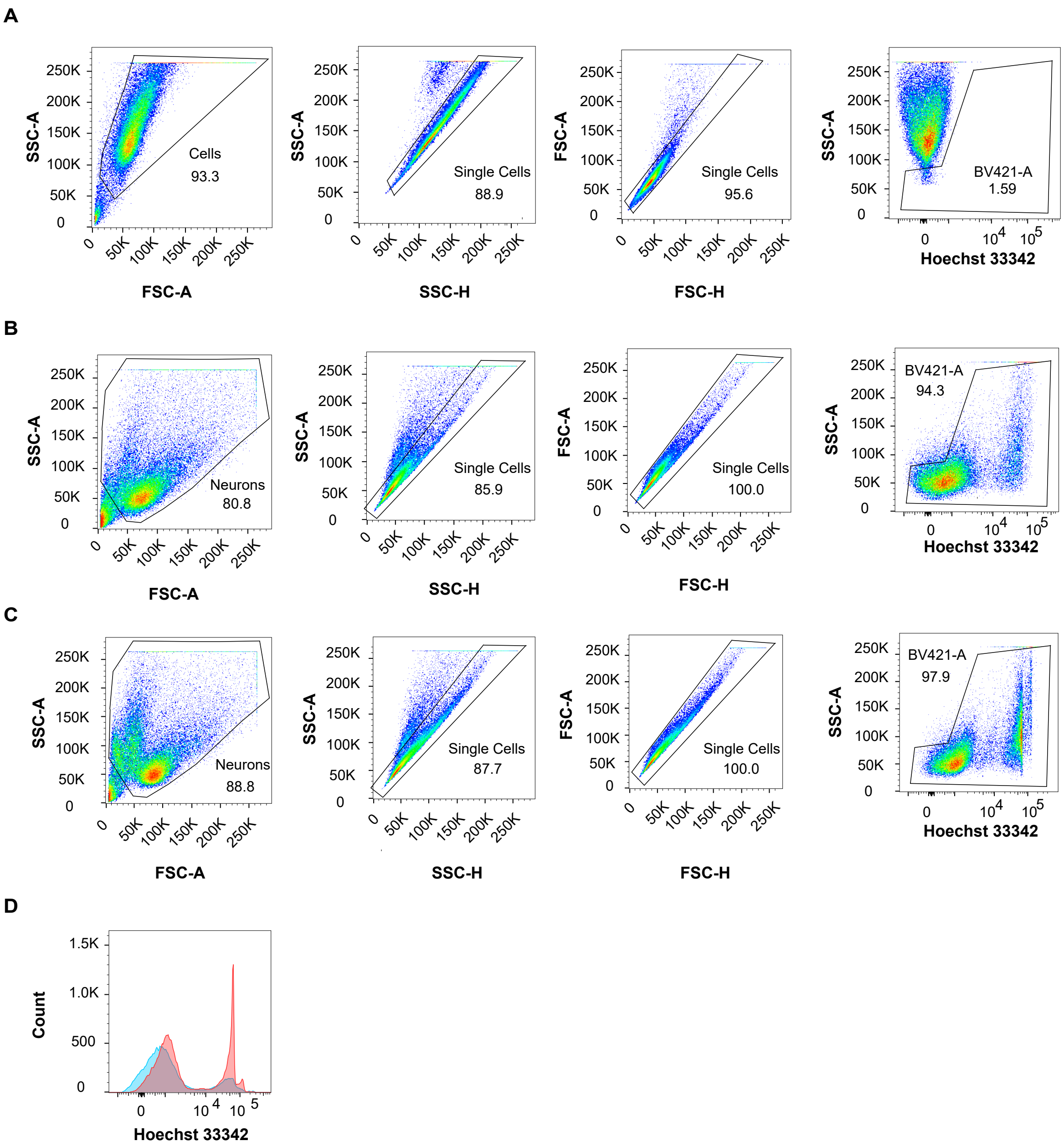

Figure S5: Gating strategy for the cell cycle analysis upon ZFHX4 KD. A) Representative gating of a dead mDAN control culture used to define the non-viable population. B) Gating strategy for the shSCRAMBLE condition. C) Gating strategy for the shZFHX4 condition. All samples were stained under the same conditions.
