# Supplementary material for: ZFHX4 is necessary for dopaminergic neuron differentiation and controls cell cycle by regulating LIN28A
Source: Stem Cell Reports. 2026 May 28;21(6):102930. doi: 10.1016/j.stemcr.2026.102930 (PMC13261933; doi:10.1016/j.stemcr.2026.102930)
Supplement: Document S2. Article plus supplemental information [file mmc9.pdf]

STEM CELL REPORTS
Article
OPEN ACCESS

# ZFHX4 is necessary for dopaminergic neuron differentiation and controls cell cycle by regulating LIN28A

Elena Valceschini,[1,5] Borja Gomez Ramos,[1,2,5] Jochen Ohnmacht,[1,2,3] Aurelien Ginolhac,[1] Marie Catillon,[1] Deborah Gerard,[1,2] Anthoula Gaigneaux,[1] Dimitrios Kyriakis,[2] Kamil Grzyb,[2] Enrico Glaab,[2] Anne Grünewald,[2] Alexander Skupin,[2] Thomas Sauter,[1] Rejko Krüger,[2,3,4] and Lasse Sinkkonen[1,6,*]
[1]Department of Health, Medicine and Life Sciences (DHML), University of Luxembourg, 4362 Belvaux, Luxembourg
[2]Luxembourg Centre for Systems Biomedicine (LCSB), University of Luxembourg, 4362 Belvaux, Luxembourg
[3]Luxembourg Institute of Health (LIH), 1445 Strassen, Luxembourg
[4]Centre Hospitalier de Luxembourg (CHL), 1210 Belair, Luxembourg
[5]These authors contributed equally
[6]Lead contact
*Correspondence: lasse.sinkkonen@uni.lu
https://doi.org/10.1016/j.stemcr.2026.102930

## SUMMARY

Parkinson's disease (PD) involves selective degeneration of midbrain dopaminergic neurons (mDANs), yet the regulatory networks governing their development remain incompletely understood. ZFHX4 has been linked to neurodevelopment across species and shows reduced expression in the PD midbrain. Through integrative analysis of our multiomic data of mDAN differentiation, we show that ZFHX4 is a super-enhancer-controlled transcription factor induced during mDAN specification. Importantly, ZFHX4 is necessary but not sufficient for mDAN differentiation. Genome-wide profiling of ZFHX4 binding revealed targeting to active promoters, and transcriptomic profiling after ZFHX4 depletion identified primary target genes enriched for cell-cycle regulation. Consistently, ZFHX4-depleted cells showed reduced proliferation and accumulated in G2 phase, impairing cell-cycle progression. LIN28A, an RNA-binding protein involved in stem-cell maintenance and microRNA maturation, is among the strongest upregulated genes upon ZFHX4 depletion, with direct ZFHX4 binding at the locus. Our findings indicate that ZFHX4 regulates mDAN maturation through a mechanism involving the LIN28A-miR-9 axis.

## INTRODUCTION

The midbrain dopaminergic neurons (mDANs) are anatomically divided into three regions, namely, substantia nigra pars compacta (SNc), ventral tegmental area (VTA), and retrorubral field (RrF). mDANs located in the SNc mainly project to the striatum and degenerate in Parkinson's disease (PD) (Björklund and Dunnett, 2007; Fahn, 2003). Notably, mDANs from the ventral tier of the SNc are known to be more susceptible to degeneration in PD compared to the ones from the dorsal tier (Damier et al., 1999). In contrast, mDANs from the VTA and RrF project to the ventral striatum and prefrontal cortex, and are involved in the control of motivation and reward (Tzschentke and Schmidt, 2000). The neurons from these areas are less affected in PD but are associated to psychiatric disorders such as schizophrenia and drug addiction (Hegarty et al., 2013; Meyer-Lindenberg et al., 2002). Understanding the molecular mechanisms behind this selective vulnerability is, therefore, crucial for identifying the causes of the disease.

Transcription factors (TFs) together with signaling molecules play a pivotal role in cellular development and specification, defining context-specific regulatory programs. TFs bind to *cis*-regulatory elements of the genome, known as enhancers, and selectively recruit co-factors and co-activators to regulate gene expression (Ong and Corces, 2011). Enhancers regulating key aspects of cell identity are often clustered into dense regions called super-enhancers (SEs), which are bound by multiple master regulators, leading to recruitment of high levels of the Mediator complex (Whyte et al., 2013). The identification of SEs typically relies on the use of methods such as chromatin immunoprecipitation sequencing (ChIP-seq) or cleavage under targets and tagmentation (CUT&Tag) to profile the occupancy of cell-type-specific TFs, histone markers or the Mediator complex (Kaya-Okur et al., 2019). SEs are generally larger in size and display higher levels of Mediator and cofactor binding (Whyte et al., 2013). These regions are particularly enriched in histone modifications such as histone H3 lysine 27 acetylation (H3K27ac), associated with active enhancers, and are characterized by exceptionally high levels of gene expression, and frequently harboring disease-associated genetic variants (Hnisz et al., 2013; Parker et al., 2013). SEs can be distinguished from typical enhancers based on a signal intensity cutoff derived from Mediator, H3K27ac, or other co-factor occupancy. Specifically, enhancers are ranked according to their ChIP-seq signal, and the cutoff is defined at the inflection point of the resulting ranked signal curve, with regions beyond this threshold classified as SE (Whyte et al., 2013). SE-controlled TFs are thus central regulators of cell fate and cell identity,


© 2026 The Author(s).
Published by Elsevier Inc. on behalf of International Society for Stem Cell Research.
This is an open access article under the CC BY license (http://creativecommons.org/licenses/by/4.0/).

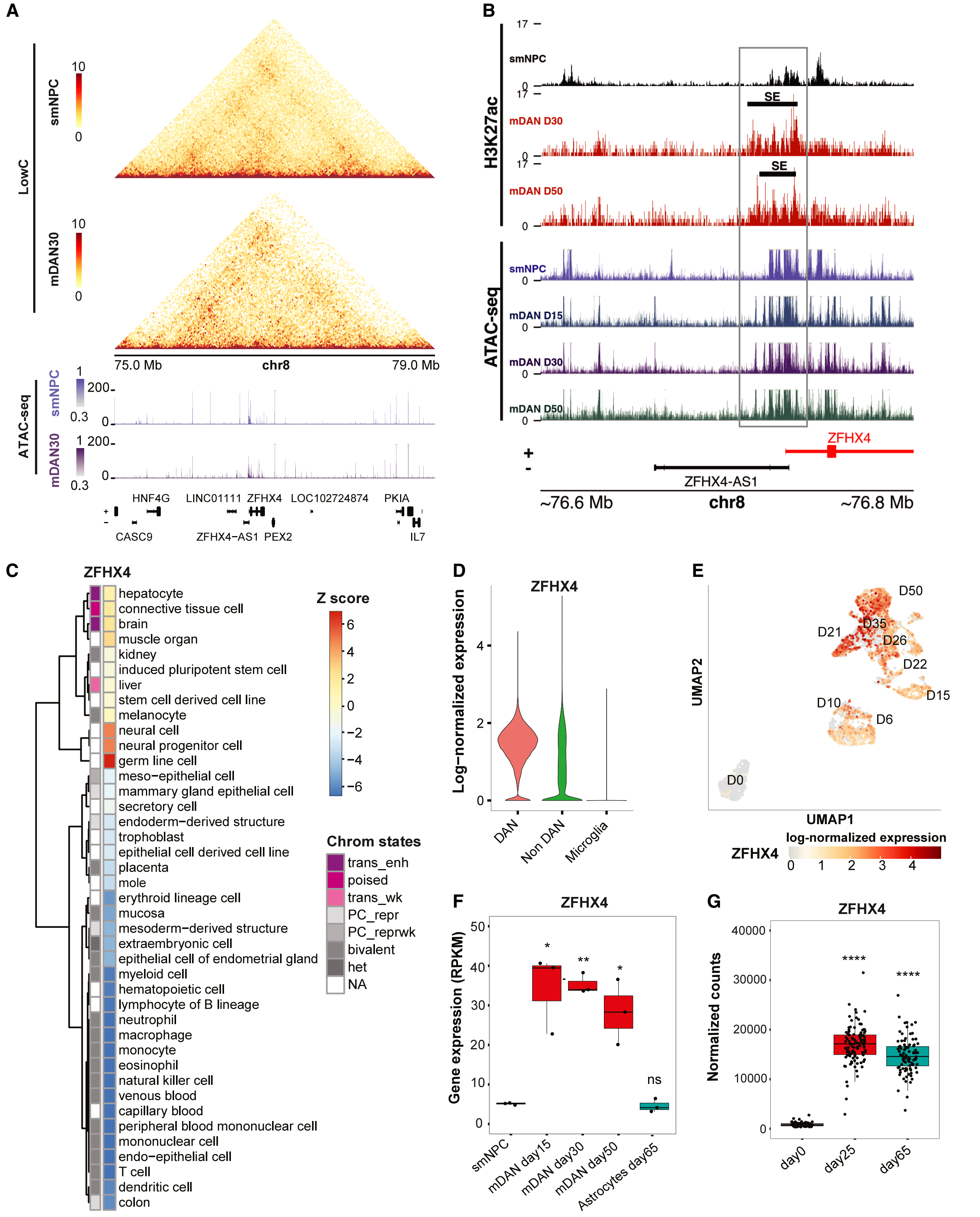


*(legend on next page)*

making their identification useful for understanding lineage specification processes.

Several TFs and morphogens controlling mDAN development have already been uncovered, including NR4A2 (also known as NURR1), one of the most prominent markers of mature mDANs, along with tyrosine hydroxylase (TH), the rate limiting enzyme for dopamine production (Arenas et al., 2015; Molinoff and Axelrod, 1971; Poulin et al., 2020; Zetterström et al., 1997). However, our current knowledge is primarily based on rodent models. To study human cells, the use of induced pluripotent stem cell (iPSC)-derived models has become fundamental, given the low number and limited accessibility of human mDANs *in vivo* (Kriks et al., 2011; Reinhardt et al., 2013). Recent studies have demonstrated the benefits of iPSC-derived dopaminergic progenitor transplants in human PD patients, showing successful mDAN recovery, lasting up to several months (Sawamoto et al., 2025; Tabar et al., 2025). Furthermore, the advent of single-cell technology has paved the way for an unbiased identification of cellular subtypes based on their gene expression, allowing to identify novel subpopulations of mDANs, including those most affected in PD (Kamath et al., 2022; La Manno et al., 2016). Therefore, deploying these technologies will enable a better reconstruction of the molecular factors driving mDAN development and aid in uncovering novel cell-based therapies (Ásgrímsdóttir and Arenas, 2020).

ZFHX4 is a TF comprising 22 zinc fingers and four homeodomains whose expression is predominant in developing human brain and muscle (Hemmi et al., 2006), as well as in mouse cartilage and limb buds (Nakamura et al., 2021). Pathogenic variants in *ZFHX4* locus were shown to correlate with aberrant brain and craniofacial development, both in human and zebrafish models (Fontana et al., 2021; Pérez Baca et al., 2025), while *Zfhx4*-deficient mice exhibit impaired endochondral ossification (Nakamura et al., 2021). Other than developmental processes, ZFHX4 has been shown to be detrimental for the survival of several types of cancers and the self-renewal of stem-cell-like cells (Ha et al., 2020; Qing et al., 2017; Zong et al., 2022). In glioblastoma tumor-initiating cells (TICs), for instance, its suppression led to an increase in neuronal markers, reduced cell proliferation, and arrest in G0/G1 state (Chudnovsky et al., 2014). In TICs ZFHX4 was found to interact with CHD4, a core member of the nucleosome remodeling complex, involved in the regulation of cell-cycle progression (Chudnovsky et al., 2014). While during palatal development, it partners with the TFs OSTERIX and RUNX2 (Nakamura et al., 2021). Furthermore, immunoprecipitation followed by mass spectrometry in neural precursor cells revealed that ZFHX4-interacting proteins are mainly involved in histone modifications, transcriptional regulation, and development (Pérez Baca et al., 2025). Taken together, these data indicate ZFHX4 as a TF interacting with members of macromolecular complexes to exert context-specific regulation of both developmental and self-renewal pathways. Here, we identified a novel role of ZFHX4 as a TF specifically induced during the *in vitro* differentiation of iPSC-derived mDANs. Depletion of ZFHX4 during neuronal differentiation led to a significant reduction in the number of mDANs, while its overexpression did not lead to any changes in mDAN count. Identification of ZFHX4 target genes using CUT&Tag profiling and transcriptomic analysis upon ZFHX4 knockdown (KD) revealed its involvement in cell-cycle regulation during mDAN differentiation, which was confirmed by immunocytochemistry (ICC) and flow cytometry. Furthermore, LIN28A, a multipotency promoting factor controlling the maturation of the neurogenesis-promoting miR-9, appeared among the most affected primary targets of ZFHX4. In summary, ZFHX4 emerged as a TF necessary for mDAN development, regulating cell cycle by controlling the expression

**Figure 1. Transcriptomic and epigenomic profile of *ZFHX4* in different models of mDANs**

(A) Topologically associated domain at *ZFHX4* locus in smNPCs and day 30 differentiated mDANs shown together with chromatin accessibility profiles at the selected time points. The ATAC-seq tracks displayed in the bottom panel correspond to the same datasets shown in (B). The tracks are displayed with a capped *y* axis; peaks exceeding the maximum plotting limit are truncated for visualization purposes. Human reference genome (GRCh38, Ensembl release 104).

(B) H3K27ac signal and chromatin accessibility at the 200-kb region flanking *ZFHX4* TSS at indicated time points of mDAN differentiation. Gray rectangle indicates the identified mDAN-specific SE.

(C) Heatmap showing the expression and the ChromGene (Jaroszewicz and Ernst, 2023) state of *ZFHX4* in 41 human tissues.

(D) Expression of *ZFHX4* in selected cell types DAN ($TH^+$, $SLC6A3^+$, $SLC18A2^+$), non-DAN ($TH^−$, $SLC6A3^−$, $SLC18A2^−$), and microglia ($TMEM119^+$, $AIF1^+$, $ITGAM^+$) from single-nuclei RNA-seq analysis of postmortem *in vivo* human SNc (Kamath et al., 2022).

(E) Uniform manifold approximation and projection showing the log-normalized expression of *ZFHX4*during time-series *in vitro* differentiation of iPSCs into mature day 50 mDANs, upon single-cell RNA-seq analysis (Novak et al., 2022).

(F) RNA-seq expression levels of *ZFHX4* during time-series differentiation from smNPCs and in smNPC-derived astrocytes ($n = 3$ independent experiments) (Gomez Ramos et al., 2024). (G) Expression of *ZFHX4* in 95 different iPSC lines differentiated into mDANs (Bressan et al., 2023). Error bars (F and G) correspond to ±1 standard deviation (SD) from the mean, *t* test, **p* value <0.05, ***p* value <0.01, *****p* value <0.0001; ns, not significant.

of targets like the miR-9-inhibiting multipotency factor LIN28A.

## RESULTS

### ZFHX4 is a super-enhancer-controlled transcriptional regulator induced during dopaminergic neurogenesis

We have previously performed transcriptomic and epigenomic profiling across a differentiation time series of iPSC-derived mDANs (Gomez Ramos et al., 2024). Integrative analysis of these datasets enabled the identification of novel SE-controlled TFs with selective expression in mDANs, as well as the reconstruction of gene regulatory networks underlying their development. Through multiple filtering steps (see STAR Methods for further details), a final list of 7 SE-controlled TFs with selective expression in mDANs was prioritized (Figure S1A). Among these putative key regulators of mDAN differentiation, ZFHX4 showed significantly decreased expression in the midbrain of PD patients compared to controls ($\log_2$-fold change = $-1.155$, $p = 0.0008$) (Tranchevent et al., 2023), making it a particularly interesting regulator for further analysis (Figure S2A).

LowC analysis of the *ZFHX4* locus revealed a >2-Mb topological domain with many regulatory interactions also from distal enhancers, carrying only one additional protein-coding gene (PEX2) and a few non-coding genes (Figure 1A) (Gérard et al., 2026). Furthermore, comparative analysis of these topological domains between small molecule-derived neural precursor cells (smNPCs) and mDANs at day 30 revealed changes in domain interactions, suggesting a reorganization of the locus during mDAN differentiation (Figure 1A). More specifically, an increase in interactions within the TAD associated with *ZFHX4* was observed in mDANs at day 30 compared to smNPCs (±1 Mb; Table S1). The progressive increase in accessibility at the *ZFHX4* locus across the differentiation time series, along with the broad enrichment of H3K27ac at its promoter, are characteristic of an SE-controlled target gene (Figure 1B). Analysis of epigenomic and transcriptomic data of 41 human tissues and cell types from EpiATLAS of the International Human Epigenome Consortium revealed a high expression of *ZFHX4* in neural cells and neural progenitors, indicating its involvement in the development and/or maintenance of neural cell identity (Figure 1C) (Bujold et al., 2016). Consistently, highly transcriptionally active chromatin state was observed only in two samples, brain and hepatocytes. Single-nuclei transcriptomic analysis of postmortem human SNc further shows a preferential expression of *ZFHX4* in mDANs compared to other cell types in the same brain region (Figure 1D) (Kamath et al., 2022). The selective expression of *ZFHX4* and induction during mDAN differentiation were also confirmed in alternative *in vitro* models of iPSC-derived mDANs through time-series analysis both at single cell (Figure 1E) and at bulk level (Figure 1F), and across tens of iPSC lines (Figure 1G) (Bressan et al., 2023; Novak et al., 2022). Together, these data suggest that ZFHX4 is an SE-controlled TF, whose function remains largely unexplored and that seems to play a selective role in the context of mDAN differentiation.

### ZFHX4 is required but not sufficient for mDAN differentiation

To assess ZFHX4 expression and cell identity during differentiation, we performed ICC for ZFHX4, TH, and the stemness marker SOX2, in smNPCs and on days 8 and 15 of mDAN differentiation (Figure S3). Quantitative analysis of ZFHX4- and TH-positive areas showed an increase from smNPCs to day 15, consistent with mDAN maturation and transcriptomic data (Figures S3A and S3B). Since TH does not stain nuclei, a direct overlap of the two proteins could not be performed. However, around half of the TH-positive cells were confirmed to co-express ZFHX4 on day 15, supporting a role for ZFHX4 during mDAN maturation (Figure S3C). Moreover, smNPCs showed the highest SOX2 mean fluorescent intensity with a reduced signal on days 8 and 15 of mDAN differentiation (Figures S3D and S3E). Finally, a fluorescence-activated cell sorting (FACS) analysis of the day 15 mDANs confirmed approximately 20% of the cells to be mCherry positive when using the TH-mCherry reporter system (TH-Rep1 cell line) that allows mCherry signal only in TH-expressing cells, as reported previously (Gomez Ramos et al., 2024).

To investigate the role of ZFHX4 in mDAN differentiation, we performed KD experiments at two selected time points of differentiation (days 1 and 9) to achieve both an early and a late KD (Figure 2A). Transduction with lentiviral vectors carrying shRNA targeting ZFHX4 resulted in an approximately 80% reduction of ZFHX4 expression in mDANs at day 15 compared to control short hairpin RNA (shRNA) (shSCRAMBLE) (Figure 2B). A consistent reduction at protein level was confirmed using ICC (Figures S4A and S4B). To study the impact of ZFHX4 depletion on mDAN numbers, neurons were analyzed using FACS on the day of analysis, taking advantage of the TH-mCherry reporter system (Gomez Ramos et al., 2024). We observed an ~60% reduction in mDANs following early KD of ZFHX4 and ~40% following late KD, suggesting ZFHX4 is required for mDAN development (Figure 2C).

To gain further insights into the role of ZFHX4 in mDAN differentiation, we overexpressed ZFHX4 following the experimental scheme shown in Figure 2A. Using CRISPR activation (CRISPRa) an almost 3-fold overexpression of ZFHX4 (Figure 2D) was achieved. However, this

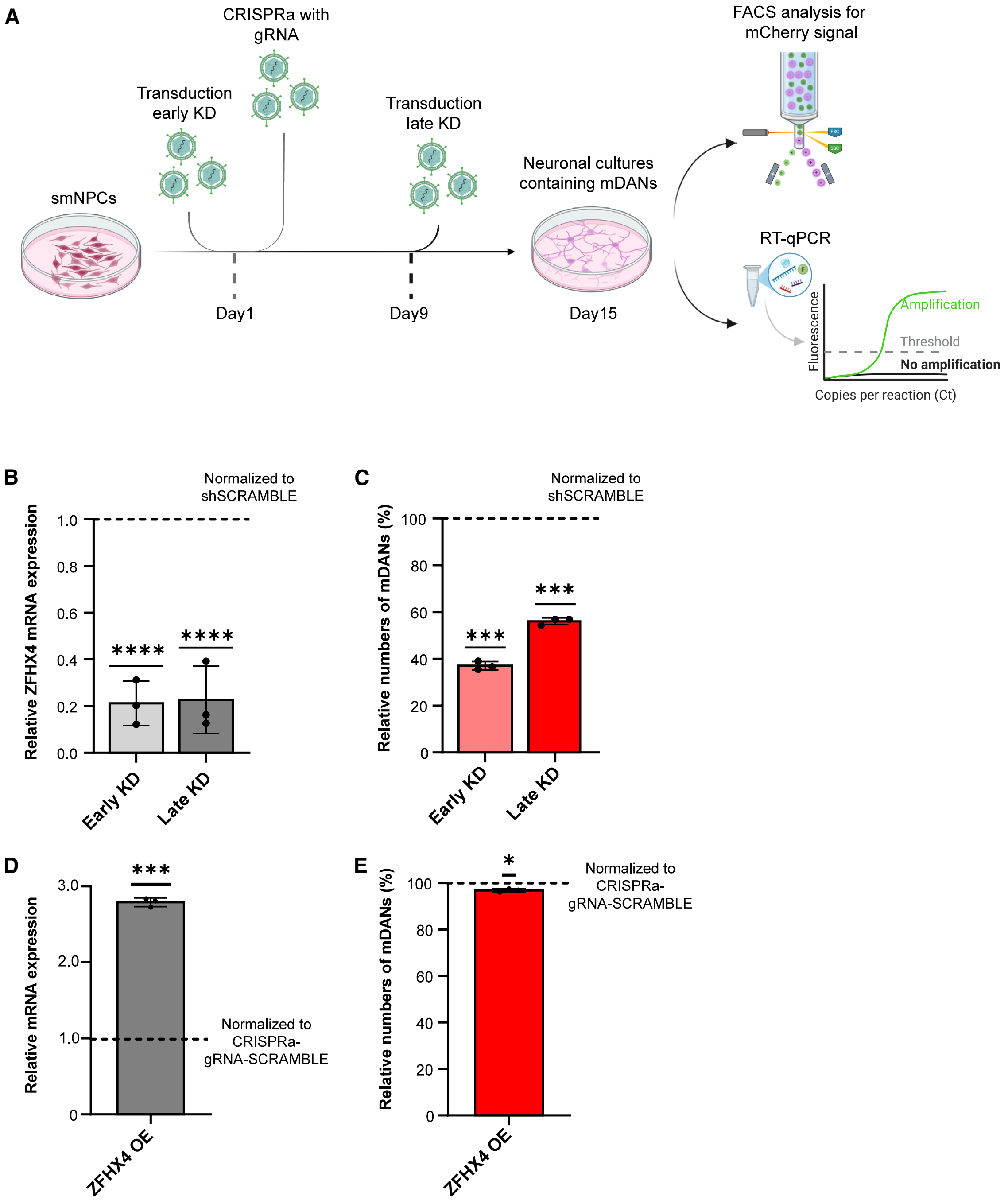


**Figure 2. ZFHX4 is necessary but not sufficient for mDAN differentiation**

(A) Schematic representation of the early and late KD experiments and the CRISPRa for overexpression. FACS and RT-qPCR analyses were conducted on day 15.
(B) Early and late transduction results showing ZFHX4 KD expression levels.
(C) Early and late transduction results showing the effect of the KD on mDAN numbers.
(D) Results of ZFHX4 overexpression levels.
(E) Effect on mDAN numbers upon ZFHX4 overexpression.
Data in all panels are representative of $n = 3$ independent experiments. Error bars (B–E) correspond to ±1 standard deviation (SD) from the mean. One-sample $t$ test was used for statistical analysis, taking 100 (C and E) or 1 (B and D) as the theoretical mean. *$p$ value <0.05, ***$p$ value <0.001, ****$p$ value <0.0001; ns, not significant.

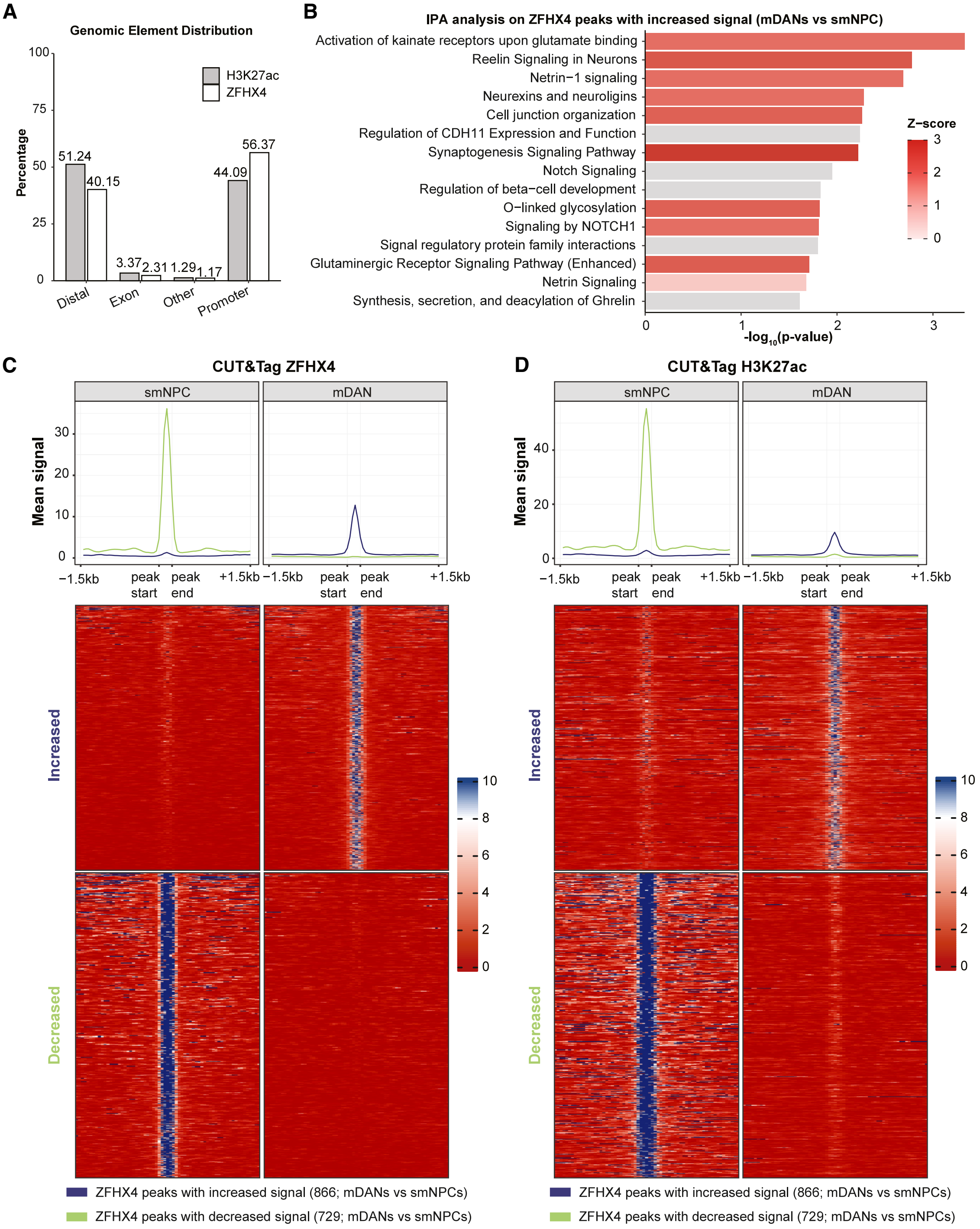


**Figure 3. ZFHX4 binds to active regulatory regions and is involved in the regulation of neuronal pathways**

(A) Genomic annotation of the 43,418 ZFHX4 called peaks and the 91,029 H3K27ac called peaks using the ChIPseeker (Yu et al., 2015) Bioconductor package with a ±3-kb transcription start site window to define the promoter region.

(B) IPA (Krämer et al., 2014) analysis of the genes associated with the 866 peaks showing increased ZFHX4 signal in day 15 mDANs compared to smNPCs (absolute $\log_2$-fold change >3.5), showing the top enriched pathways ranked by $-\log_{10}(p \text{ value})$ with colors indicating predicted pathway *Z* score.

*(legend continued on next page)*

perturbation did not result in any changes in the proportion of mDANs (Figure 2E). Altogether, these results indicate that ZFHX4 is required for mDAN development, but not sufficient on its own to drive their differentiation.

### ZFHX4 binds preferentially to active promoters and controls genes involved in cell-cycle regulation

To map the putative targets of ZFHX4, we performed CUT&Tag analysis for ZFHX4 binding and H3K27ac modifications in smNPCs and in FACS-sorted mCherry-expressing mDANs. The mean fragments in peaks score for both antibodies was lower in mDANs, most likely reflecting differences between the cell types and higher uniformity of the stem-cell culture that does not require FACS-assisted sorting of the cells. A total of 43,418 ZFHX4 peaks and 91,029 H3K27ac peaks was detected for both cell types (Table S2). Genomic annotation of the called peaks showed a more frequent enrichment for ZFHX4 at gene promoters and transcription start sites compared to H3K27ac. Conversely, ZFHX4 exhibited lower enrichment at distal regulatory regions (Figure 3A). To better understand the regions targeted by ZFHX4, we identified peaks with differential ZFHX4 binding in mDANs relative to smNPCs (absolute $\log_2$-fold change >3.5, false discovery rate [FDR] <0.05) (Table S2). This analysis identified 866 peaks with increased signal and 729 with decreased signal in mDANs. Ingenuity Pathway Analysis (IPA, Qiagen) (Krämer et al., 2014) of the genes in proximity of 866 peaks exhibiting increased signal in mDANs relative to smNPCs showed an enrichment of pathways associated to neuronal and synaptic signaling processes (Figure 3B). The differential peaks that gained or lost ZFHX4 binding in mDANs are ranked based on differential ZFHX4 signal and visualized as a heatmap in Figure 3C. Interestingly, visualizing the H3K27ac signal at the same regions revealed a comparable gain or loss of acetylation at the ZFHX4-bound sites, suggesting that ZFHX4 is largely associated with active regulatory regions (Figure 3D).

To further analyze the downstream targets of ZFHX4, we performed RNA sequencing (RNA-seq) analysis following the late shRNA-induced KD of ZFHX4 in mDANs, identifying 1,947 differentially expressed genes (DEGs) between shZFHX4 and shSCRAMBLE (absolute $\log_2$-fold change >0.5, FDR <0.05), with comparable number of up- and downregulated genes (Figure 4A; Table S3). Gene set enrichment analysis (GSEA) of the DEGs using the Reactome Database (Jassal et al., 2020; Milacic et al., 2024) revealed an enrichment of pathways related to the cell cycle, particularly S phase and transition from mitotic (M) phase to G1 phase (Figure 4B). To identify putative primary targets of ZFHX4, we intersected the list of DEGs with the CUT&Tag analysis-derived 14,451 ZFHX4-bound targets annotated to promoter regions (±3 kb from the TSS [transcription start site]; Figure 4C; Table S4). IPA (Krämer et al., 2014) analysis of these candidate primary targets revealed enrichment for pathways related to cell-cycle regulation, including cell-cycle checkpoints and mitotic progression (Figure 4D). Together, these data suggest a role of ZFHX4 in the regulation of the cell-cycle dynamics during mDAN development.

### ZFHX4 depletion leads to cell-cycle alterations in differentiating neuronal progenitors

Based on the observation that ZFHX4 appears to regulate cell-cycle genes during mDAN differentiation, we hypothesized that ZFHX4 might be involved in the completion of the cell cycle required for cell-cycle exit during neurodevelopment, facilitating the transition from a proliferative state to a post-mitotic, quiescent state (Thomaidou et al., 2010). We, therefore, analyzed the proliferation state of the cells upon ZFHX4 KD.

First, to assess the role of ZFHX4 in cell division during mDAN differentiation, we performed a live-cell imaging-based proliferation assay from days 2 to 8 of differentiation, following early ZFHX4 KD. While growth rates were comparable at early time points, cells showed a progressive and significant reduction in confluency starting from day 6, coinciding with the onset of ZFHX4 expression induction, suggesting a role of ZFHX4 in sustaining cell expansion during mDAN differentiation (Figure 5A).

Next, we stained the cells on day 8 of differentiation for the proliferation marker Ki67, the mitosis marker phospho-histone H3 (PH3), and the neuronal marker MAP2. Upon ZFHX4 KD, we observed an increasing trend for both Ki67 and PH3 staining, suggesting a rise in the number of cells remaining in cell cycle when ZFHX4 was depleted, but this increase was not found to be statistically significant (Figure S4).

Finally, we performed flow cytometry to further investigate the role of ZFHX4 in cell cycle regulation. Early ZFHX4 KD was confirmed on the day of analysis by quantitative reverse-transcription PCR (RT-qPCR), reaching approximately 50% reduction in expression (Figure 5B).

(C) Heatmaps and profiles of ZFHX4 CUT&Tag signal in smNPCs and day 15 mDANs at the 866 peaks showing increased ZFHX4 signal in mDANs compared to smNPCs and the 729 peaks showing decreased ZFHX4 signal. Data represent the mean CUT&Tag signal from 4 to 3 independent replicates in smNPCs and mDANs, respectively.
(D) Heatmaps and profiles of H3K27ac CUT&Tag signal in smNPCs and day 15 mDANs at the same set of regions ranked as in (C). Data represent mean CUT&Tag signal from 4 to 3 independent replicates in smNPCs and mDANs, respectively.

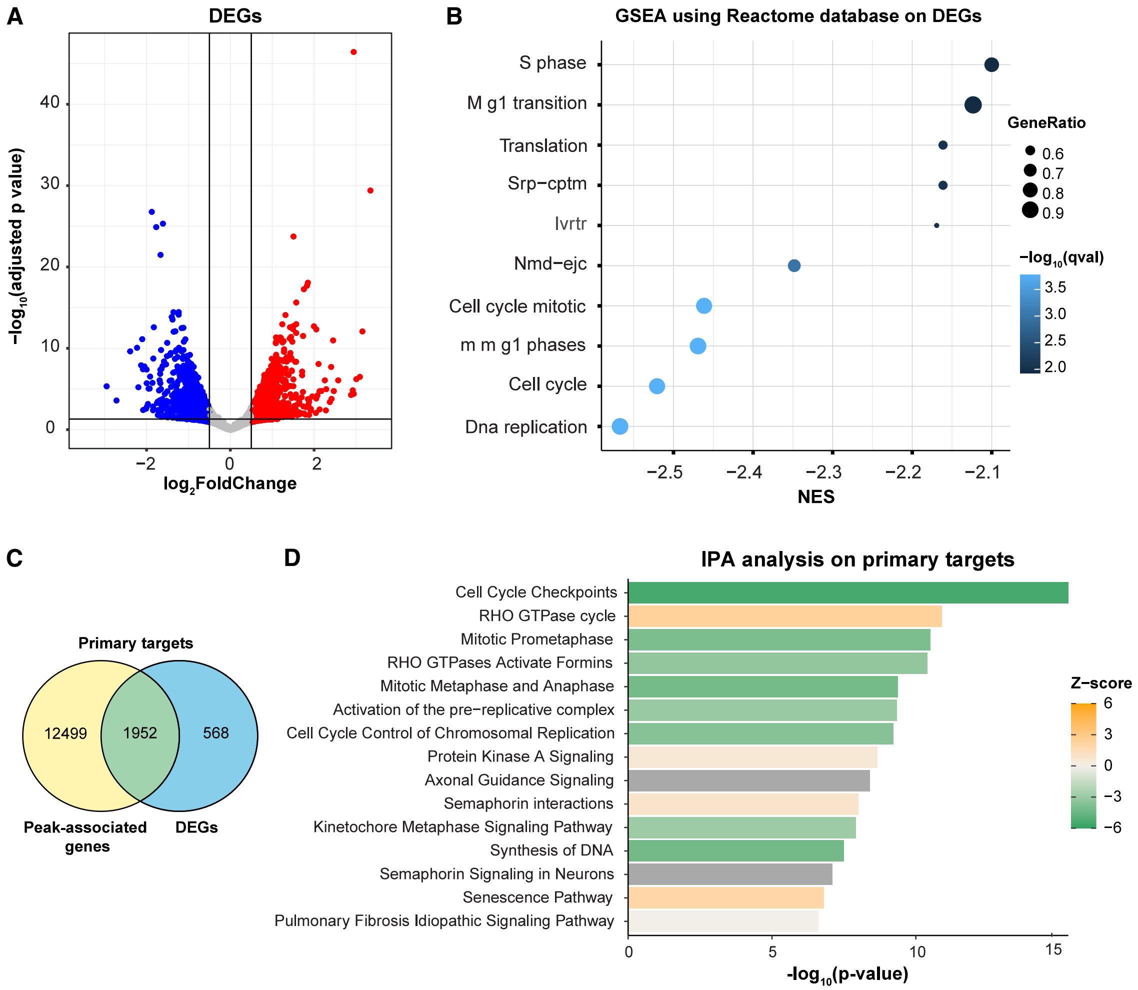


**Figure 4. ZHFX4 is involved in the regulation of the cell cycle**

(A) Volcano plot of the 1,947 significant DEGs from the RNA-seq analysis of neurons on day 15 following a late transduction with shScramble or shZFHX4. Black lines represent cutoff according to FDR <0.05 and absolute $\log_2$-fold change >0.5. Red dots represent the upregulated genes and blue dots, the downregulated genes.

(B) GSEA enrichment analysis of the 1,947 DEGs using the Reactome Database. *x* axis represents the normalized enrichment score (NES).

(C) Venn diagram showing the overlap between the 14,451 genes annotated from promoter-associated peaks identified by ZFHX4 CUT&Tag and the 2,520 DEGs (FDR <0.05), resulting in the identification of 1,952 putative ZFHX4 primary targets.

(D) IPA (Krämer et al., 2014) of the 1,952 identified primary targets, showing the top enriched pathways ranked by $-\log_{10}(p \text{ value})$ and color representing predicted *Z* score.

Staining with the DNA-binding fluorescent dye Hoechst 33342 revealed significant changes in the proportion of cells in the different cell-cycle phases following ZFHX4 depletion, without corresponding changes in overall cell viability (Figures 5C and 5D). Specifically, there was an increase in the number of cells in G2 phase and a corresponding decrease in G1 phase cells, indicating a failure to exit and accumulation in the later stages of the cell cycle in the absence of ZFHX4 (Figure 5E). Altogether, these data suggest that ZFHX4 is involved in regulating the cell cycle during mDAN development; specifically, in the absence of ZFHX4, cells exhibit reduced growth rates and accumulate in G2 phase.

## ZFHX4 is necessary for repression of LIN28A and induction of neurogenic miR-9 during mDAN differentiation

To further characterize the downstream targets of ZFHX4, we analyzed the most significantly upregulated and downregulated genes following ZFHX4 KD. Among the most upregulated genes, we identified LIN28A (7.69-fold increase), while the primary microRNA (pri-miRNA) miR-9-3 was among the

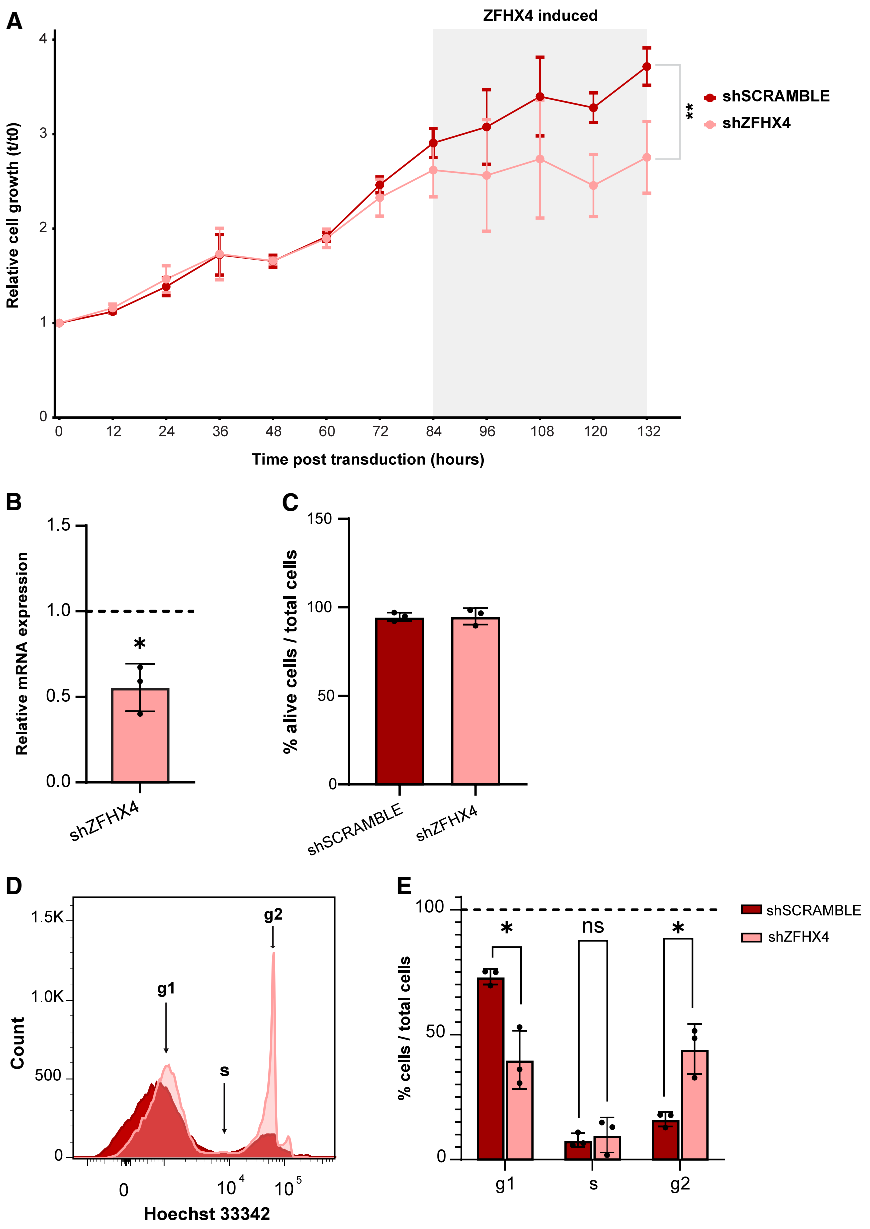


**Figure 5. Altered cell cycle upon ZFHX4 depletion**

(A) Cell proliferation assay measured every 12 h starting from 1 day post-transduction (corresponding to day 2 of differentiation) and continued until day 8 of differentiation. Data are represented as relative cell growth normalized to $t_0$. Error bars correspond to ±1 standard error of the mean (SEM); paired *t* test at time points 84–132 h, corresponding to the ZFHX4 window of induced expression (gray area), ns, not significant (*n* = 3 independent experiments).

(B) Expression level of ZFHX4 after early KD and RT-qPCR analysis on day 8 (*n* = 3 independent experiments).

(C) Percentage of viable cells quantified by flow cytometry following staining with Hoechst 33342 for shSCRAMBLE and shZFHX4. Dead cells were used to establish the gating strategy.

(D) Hoechst 33342-positive cell counts after flow cytometry for either shZFHX4 (red) or shSCRAMBLE (pink). Analysis was done on day 8 upon early ZFHX4 KD (*n* = 3 independent experiments).

(E) Quantification of cell counts according to each cell-cycle phase between shSCRAMBLE and shZFHX4 (*n* = 3 independent experiments).

Error bars (B and E) correspond to ±1 standard deviation (SD) from the mean; *t* test, **p* value <0.05, ***p* value <0.01; ns, not significant.

most downregulated genes (4.3-fold decrease) (Figure 6A). LIN28A is an RNA-binding protein known to promote pluri- and multipotency and has been previously shown to participate in an inhibitory loop with the neurogenesis-promoting miR-9 (Nowak et al., 2014). We, thus, explored the transcriptomic and epigenomic profiles of these two factors in detail in our *in vitro* models (Figures 6B and 6C).

Time-series RNA-seq analysis confirmed that *LIN28A* is highly expressed in smNPCs and is progressively repressed during mDAN differentiation, whereas the neurogenic *pri-miR-9-3* is induced during mDAN development (Figure 6B). ATAC-seq analysis of the *LIN28A* locus showed a progressive decrease in chromatin accessibility, consistent with its transcriptional repression during mDAN differentiation (Figure 6C). This reduction in accessibility was concomitant with decreased acetylation, as shown by CUT&Tag analysis in smNPCs relative to day 15 mDANs. Interestingly, CUT&Tag analysis revealed strong ZFHX4 enrichment at the *LIN28A* promoter, with an increased signal at an upstream regulatory region during mDAN development, suggesting direct transcriptional regulation of *LIN28A* by ZFHX4 (Figure 6C). We then analyzed the expression of *LIN28A*, *TH*, *ZFHX4*, and mature miR-9 at days 0, 4, 6, 8, and 16 of early mDAN differentiation. The results

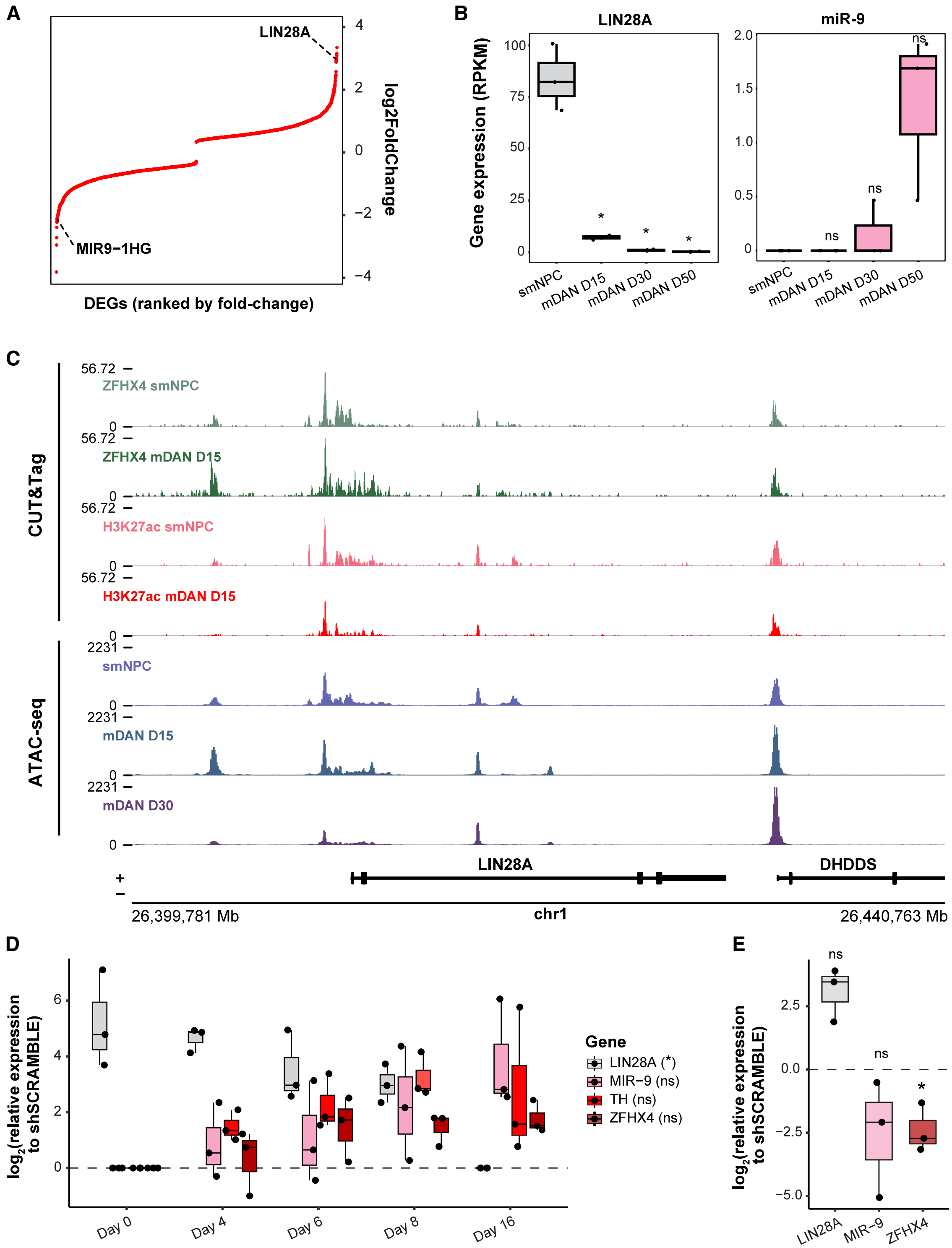


**Figure 6. Differentiation failure is associated with ineffective repression of LIN28A**

(A) Rank plot of the 2,520 DEGs upon ZFHX4 KD highlighting LIN28A and miR-9 position.

(B) RNA-seq expression levels of LIN28A and pri-miR-9-3 during mDAN time-series differentiation ($n$ = 3 independent experiments).

(C) CUT&Tag profiles of ZFHX4 and H3K27ac at *LIN28A* locus in smNPC and in day 15 mDANs. ATAC-seq profiles at selected time points of *LIN28A* locus.

*(legend continued on next page)*

confirmed the transcriptional repression of *LIN28A* during mDAN differentiation, concurrent with the induction of *TH*, *ZFHX4*, and miR-9 (Figure 6D). Finally, we validated the effects of ZFHX4 depletion using TaqMan microRNA assays and RT-qPCR, indicating an increase of LIN28A and the downregulation of mature miR-9 (Figure 6E). Altogether, these data indicate a role of ZFHX4 in the regulation of the LIN28A-miR-9 loop. Specifically, ZFHX4 appears to downregulate *LIN28A*, possibly directly at the transcriptional level, leading to its depletion during normal mDAN differentiation, in turn allowing the induction of neurogenesis-promoting miRNA miR-9, further supporting the suppression of cell cycle (Coolen et al., 2012, 2013).

## DISCUSSION

In this study, we identified ZFHX4 as a novel SE-controlled TF regulating mDAN differentiation. While ZFHX4 has previously been linked to neurodevelopmental processes, its role in mDAN development has not been described before. Mutations in the ZFHX4 locus have been associated with intellectual disability as well as craniofacial and brain developmental defects (Pérez Baca et al., 2025). ZFHX4 expression has also been observed in cartilage, where it has been demonstrated to play a role during endochondral ossification, suggesting its importance in developmental regulation (Nakamura et al., 2021). We have performed an integrative analysis of ATAC-seq, RNA-seq, and low-input ChIP-seq data of *in vitro* iPSC-derived mDANs, identifying several SE-controlled TFs involved in mDAN differentiation (Gomez Ramos et al., 2024). Among these, ZFHX4 emerged as a newly associated TF, with preferential expression in mDANs and reduced expression *in vivo* in PD. Functional studies revealed that ZFHX4 depletion during mDAN differentiation leads to a significant reduction in the proportion of mDANs, suggesting a necessary role during their development (Figures 2B and 2C). However, ZFHX4 overexpression was not sufficient to increase the number of mDANs (Figures 2D and 2E). The complex structure of ZFHX4 comprising 4 homeodomains and 22 zinc fingers suggests that it interacts with multiple co-factors to mediate context-specific regulatory functions. Immunoprecipitation coupled with mass spectrometry has identified ZFHX4-associated co-factors in different biological systems, including neural precursor cells and TICs (Chudnovsky et al., 2014; Pérez Baca et al., 2025). These findings support the notion that ZFHX4 requires multiple co-factors to exert context-specific roles. Therefore, overexpression on its own is probably not sufficient to increase the number of mDANs during differentiation.

Transcriptomic analysis following ZFHX4 KD revealed enrichment of cell-cycle-related pathways in mDANs, particularly those involved in the S phase and the transition from M phase to G1 phase. Putative primary targets of ZFHX4, identified through CUT&Tag and RNA-seq analysis, were enriched for E2F target genes and components of the NF-Y TF complex (Table S4). The cell cycle is a tightly regulated process, with distinct checkpoints controlled by multiple factors to ensure proper cell division. E2Fs are a large family of TFs whose activity depends on the phosphorylation state of retinoblastoma proteins (pRB) and which sequentially regulate the expression of cell-cycle-related genes during cell division (Kent and Leone, 2019). NF-Y is a heterotrimeric TF complex that modulates the expression of genes involved in cell proliferation and developmental processes (Dolfini et al., 2025). During neurodevelopment, cells transition from a multipotent, proliferative state into a non-proliferative, quiescent state referred to as the G0 phase, making their exit from the cell-cycle division (Thomaidou et al., 2010). The involvement of ZFHX4 in cell-cycle regulation has previously been demonstrated in the context of glioma TICs where ZFHX4 suppression led to a decrease in the proliferation and arrest in G0/G1 state (Chudnovsky et al., 2014). However, ZFHX4 deficiency in both mouse and zebrafish models resulted in a failure to differentiate into mature states (Nakamura et al., 2021; Pérez Baca et al., 2025). Moreover, in the context of neural differentiation, ZFHX4 has been shown to bind to TFs that promote cellular proliferation (Pérez Baca et al., 2025). Nevertheless, the regulation of the cell cycle during neurodevelopment, as well as the specific role of ZFHX4 during developmental processes, remains incompletely understood. We thus hypothesized that ZFHX4 may facilitate the transition from proliferative progenitor cells to mature neurons during mDAN differentiation. Indeed, a live-cell imaging confirmed reduced proliferation of cells undergoing mDAN differentiation upon depletion of ZFHX4 (Figure 5A). A more detailed analysis of the cell cycle state following ZFHX4 KD did not produce significant changes in the proliferation marker Ki67 or in the mitotic marker PH3, although a trend toward increased

(D) $Log_2$-transformed expression levels of LIN28A, TH, ZFHX4, miR-9 after RT-qPCR and TaqMan during time-series mDAN differentiation at days 0, 4, 6, 8, and 16 ($n = 3$ independent experiments).
(E) Expression levels of LIN28A, ZFHX4, and miR-9 upon late KD of ZFHX4 and analysis on day 15 ($n = 3$ independent experiments). Boxplots represent the median (center line), interquartile range (box), and minimum-maximum values (whiskers), with individual data points overlaid. Statistical analysis: *t* test (B and E), one-way ANOVA (D); *$p$ value <0.05; ns, not significant.

Ki67 was observed (Figures S4A–S4D). Ki67 is a DNA-binding protein that displays distinct subnuclear localizations throughout all active phases of the cell cycle and is absent only in the G0 phase. However, it does not distinguish between G1, S, and G2/M phases (Sun and Kaufman, 2018). In contrast, PH3 marks a post-translational modification of H3 that occurs during the late G2 phase and mitosis, disappearing by anaphase, and therefore, does not label cells in the G1 or S phase (Kim et al., 2017). We thus decided to perform flow cytometry analysis using Hoechst 33342, a DNA fluorescent dye that enables discrimination of the G1, S, G2 phases based on the DNA content. The analysis revealed an increase in the number of cells in the G2 phase and a corresponding decrease in the G1 phase cells following ZFHX4 KD (Figures 4E–4G). These findings indicate that in the absence of ZFHX4, cells fail to exit the cell cycle and instead accumulate in late stages of cell cycle, thereby impairing proper development.

To further investigate the underlying mechanisms connecting ZFHX4 to mDAN differentiation and cell cycle control, we examined the putative primary targets of ZFXH4 based on our transcriptomic and epigenomic data. LIN28A was among the most upregulated genes upon ZFHX4 depletion (Figure 6), and its promoter region was enriched for ZFHX4 signal in CUT&Tag, suggesting a direct transcriptional regulation by ZFHX4. The RNA-binding protein LIN28A is a known reprogramming factor implicated in early embryonic development and pluripotency (Yu et al., 2007). LIN28A participates in the post-transcriptional regulation of miRNA processing. Notably, during neurodevelopment, LIN28A is known to inhibit the maturation of miR-9, an miRNA that in turn promotes neurogenesis. Consistently, miR-9 expression was reduced (Figures 6B–6E) upon ZFHX4 KD, supporting a regulatory axis involving ZFHX4, LIN28A, and miR-9 (Nowak et al., 2014). Hence, the strong upregulation of LIN28A and the presence of ZFHX4 at *LIN28A* locus, together with its role in maintaining pluripotency, supports the hypothesis that ZFHX4 contributes to silencing of multipotency and proliferative programs, and promotes neurodevelopment.

In conclusion, we have identified a novel role for ZFHX4 as an SE-controlled TF essential for human mDAN development. ZFHX4 appears to be regulating the cell cycle by inhibiting multipotency programs, specifically by allowing repression of the pluri- and multipotency-promoting factor LIN28A. This releases the expression of miR-9, promotes neurodevelopment, and activates postmitotic programs.

## RESOURCE AVAILABILITY

### Lead contact

Requests for further information and resources should be directed to and will be fulfilled by the lead contact, Lasse Sinkkonen (lasse.sinkkonen@uni.lu).

### Materials availability

This study did not generate new unique reagents.

### Data and code availability

- Gene expression data from PD case-control brain transcriptomics study were obtained from the Gene Expression Omnibus (GEO) under accession number GSE8397 (Tranchevent et al., 2023).
- Gene expression data from single-nucleus RNA-seq analysis of human SNc (Kamath et al., 2022) were obtained from the Broad Institute Single Cell Portal (SCP1768 and SCP1769) and are publicly available via GEO under accession number GSE178265. Raw sequence-level data are available through dbGaP (phs002879.v1.p12).
- Data used in the preparation of this article were obtained on May 22, 2023, from the Parkinson's Progression Markers Initiative (PPMI) database (www.ppmi-info.org/access-data-specimens/download-data), RRID:SCR_006431. For up-to-date information on the study, visit http://www.ppmi-info.org.
- The source RNA-seq, ATAC-seq, and ChIP-seq fastq files (Gomez Ramos et al., 2024) are available at https://ega-archive.org/, under the accession number EGAD00001009288. The LowC data are available under the accession number EGAC00001002822.
- The newly generated RNA-seq and CUT&Tag fastq files have been deposited at https://ega-archive.org/, under the accession number EGAD50000001605. Additional intermediate files can be provided upon request.
- The code used for the single-nuclei RNA-seq, RNA-seq, and CUT&Tag data analysis as well as the MATLAB code used to analyze images from the high-content imaging analysis and the codes for figure generation are available at https://github.com/sysbiolux/Valceschini_et_al_2025. The Snakemake used in this study for the RNA-seq analysis is available at https://gitlab.com/uniluxembourg/fstm/dlsm/bioinfo/snakemake-rna_seq v0.2.3.


## ACKNOWLEDGMENTS

The computational analysis presented in this paper were carried out using the HPC facilities of the University of Luxembourg. We thank the Bioimaging Platform of the Luxembourg Center for Systems Biomedicine for their support. All schematic representations were created with BioRender.com. E.V. and L.S. were supported by the AUDACITY grant from the Institute of Advanced Studies at the University of Luxembourg (GENERIC). This work was supported by the Luxembourg National Research Fund within the National Center of Excellence in Research on Parkinson's Disease (NCER-PD; FNR/NCER13/BM/11264123) and the PEARL program (FNR/P13/6682797 to R.K.). B.G.R. was funded by the Luxembourg National Research Fund through the PARK-QC doctoral training unit (PRIDE17/12244779/PARK-QC).; L.S., B.G.R., J.O., and D.G. have received funding from Fondation du Pélican de Mie et Pierre Hippert-Faber and Luxembourg Rotary Foundation. PPMI, a public-private partnership, is funded by the Michael J. Fox Foundation for Parkinson's Research and funding partners, including 4D

Pharma, Abbvie, AcureX, Allergan, Amathus Therapeutics, Aligning Science Across Parkinson's, AskBio, Avid Radiopharmaceuticals, BIAL, Biogen, Biohaven, BioLegend, BlueRock Therapeutics, Bristol-Myers Squibb, Calico Labs, Celgene, Cerevel Therapeutics, Coave Therapeutics, DaCapo Brainscience, Denali, Edmond J. Safra Foundation, Eli Lilly, Gain Therapeutics, GE HealthCare, Genentech, GSK, Golub Capital, Handl Therapeutics, Insitro, Janssen Neuroscience, Lundbeck, Merck, Meso Scale Discovery, Mission Therapeutics, Neurocrine Biosciences, Pfizer, Piramal, Prevail Therapeutics, Roche, Sanofi, Servier, Sun Pharma Advanced Research Company, Takeda, Teva, UCB, Vanqua Bio, Verily, Voyager Therapeutics, the Weston Family Foundation, and Yumanity Therapeutics. The transcriptomic and epigenomic data supporting the conclusions of this article are available in the IHEC EpiATLAS resource repository, https://epigenomesportal.ca/ihec/, (International Human Epigenome Consortium, EpiATLAS, a reference for human epigenomic research, in preparation).

## AUTHOR CONTRIBUTIONS

Conceptualization, E.V., B.G.R., R.K., and L.S.; methodology, E.V., B.G.R., J.O., A. Ginolhac, M.C., D.G., A. Gaigneaux, D.K., K.G., A. Grünewald, and A.S.; investigation, E.V., B.G.R., J.O., E.G., and L.S.; writing – original draft, E.V. and L.S.; visualization, E.V., B.G.R., A. Ginolhac, and E.G.; supervision, T.S., R.K., and L.S.; funding acquisition J.O., D.G., R.K., and L.S.

## DECLARATION OF INTERESTS

The authors declare no conflict of interest.

## DECLARATION OF GENERATIVE AI AND AI-ASSISTED TECHNOLOGIES IN THE WRITING PROCESS

The authors used ChatGPT (OpenAI, GPT-4 architecture) to assist with refinement of analysis code and language editing of the manuscript. The AI tool was used under full author supervision. All outputs were critically reviewed, validated, and modified as necessary by the authors. The authors take full responsibility for the content of the manuscript.

## STAR★METHODS

Detailed methods are provided in the online version of this paper and include the following:

- KEY RESOURCES TABLE
- EXPERIMENTAL MODEL AND STUDY PARTICIPANT DETAILS
  - Cell lines
  - Ethics statement
- METHOD DETAILS
  - Cell culture and differentiation
  - Bacterial culture, plasmid extraction and lentivirus production
  - Transduction
  - Flow cytometry and FACS
  - Total RNA extraction, cDNA synthesis and RT-qPCR
  - TaqMan assay
  - CRISPR activation
  - Live-cell imaging-based proliferation assay
  - Immunocytochemistry
  - High-content imaging-based time-course analysis of mDAN differentiation
  - High-content imaging proliferation assay following ZFHX4 KD
  - Sequencing
  - RNA-seq data analysis
  - Identification of SE-controlled transcription factors
  - Low chromosome conformation capture (LowC)
  - LowC data analysis
  - CUT&Tag
  - CUT&Tag data analysis
  - Transcriptomic analysis of lateral SNc in PD
  - Analysis of single-nuclei RNA-seq of human SNc
  - Oligonucleotides
- QUANTIFICATION AND STATISTICAL ANALYSIS

## SUPPLEMENTAL INFORMATION

Supplemental information can be found online at https://doi.org/10.1016/j.stemcr.2026.102930.

Received: July 28, 2025
Revised: April 25, 2026
Accepted: April 29, 2026
Published: May 28, 2026

## REFERENCES

Arenas, E., Denham, M., and Villaescusa, J.C. (2015). How to make a midbrain dopaminergic neuron. Development *142*, 1918–1936. https://doi.org/10.1242/dev.097394.

Ásgrímsdóttir, E.S., and Arenas, E. (2020). Midbrain Dopaminergic Neuron Development at the Single Cell Level: In vivo and in Stem Cells. Front. Cell Dev. Biol. *8*, 463. https://doi.org/10.3389/fcell.2020.00463.

Björklund, A., and Dunnett, S.B. (2007). Dopamine neuron systems in the brain: an update. Trends Neurosci. *30*, 194–202. https://doi.org/10.1016/j.tins.2007.03.006.

Bressan, E., Reed, X., Bansal, V., Hutchins, E., Cobb, M.M., Webb, M.G., Alsop, E., Grenn, F.P., Illarionova, A., Savytska, N., et al. (2023). The Foundational Data Initiative for Parkinson Disease: Enabling efficient translation from genetic maps to mechanism. Cell Genom. *3*, 100261. https://doi.org/10.1016/j.xgen.2023.100261.

Bujold, D., Morais, D.A.d.L., Gauthier, C., Côté, C., Caron, M., Kwan, T., Chen, K.C., Laperle, J., Markovits, A.N., Pastinen, T., et al. (2016). The International Human Epigenome Consortium Data Portal. Cell Syst. *3*, 496–499.e2. https://doi.org/10.1016/j.cels.2016.10.019.

Chudnovsky, Y., Kim, D., Zheng, S., Whyte, W.A., Bansal, M., Bray, M.-A., Gopal, S., Theisen, M.A., Bilodeau, S., Thiru, P., et al. (2014). ZFHX4 Interacts with the nurd Core Member CHD4 and Regulates the Glioblastoma Tumor-Initiating Cell State. Cell Rep. *6*, 313–324. https://doi.org/10.1016/j.celrep.2013.12.032.

Coolen, M., Thieffry, D., Drivenes, Ø., Becker, T.S., and Bally-Cuif, L. (2012). Mir-9 Controls the Timing of Neurogenesis through the Direct Inhibition of Antagonistic Factors. Dev. Cell *22*, 1052–1064. https://doi.org/10.1016/j.devcel.2012.03.003.

Coolen, M., Katz, S., and Bally-Cuif, L. (2013). Mir-9: a versatile regulator of neurogenesis. Front. Cell. Neurosci. *7*, 220. https://doi.org/10.3389/fncel.2013.00220.

Damier, P., Hirsch, E.C., Agid, Y., and Graybiel, A.M. (1999). The substantia nigra of the human brain. II. Patterns of loss of dopamine-containing neurons in Parkinson's disease. Brain *122*, 1437–1448. https://doi.org/10.1093/brain/122.8.1437.

Dobin, A., Davis, C.A., Schlesinger, F., Drenkow, J., Zaleski, C., Jha, S., Batut, P., Chaisson, M., and Gingeras, T.R. (2013). STAR: ultrafast universal RNA-seq aligner. Bioinformatics *29*, 15–21. https://doi.org/10.1093/bioinformatics/bts635.

Dolfini, D., Imbriano, C., and Mantovani, R. (2025). The role(s) of NF-Y in development and differentiation. Cell Death Differ. *32*, 195–206. https://doi.org/10.1038/s41418-024-01388-1.

Fahn, S. (2003). Description of Parkinson's Disease as a Clinical Syndrome. Ann. N. Y. Acad. Sci. *991*, 1–14. https://doi.org/10.1111/j.1749-6632.2003.tb07458.x.

Fontana, P., Ginevrino, M., Bejo, K., Cantalupo, G., Ciavarella, M., Lombardi, C., Maioli, M., Scarano, F., Costabile, C., Novelli, A., and Lonardo, F. (2021). A ZFHX4 mutation associated with a recognizable neuropsychological and facial phenotype. Eur. J. Med. Genet. *64*, 104321. https://doi.org/10.1016/j.ejmg.2021.104321.

Gérard, D., Ohnmacht, J., Gomez-Ramos, B., Catillon, M., Sharif, J., Baumgarten, N., Hecker, D., Ginolhac, A., Landoulsi, Z., Valceschini, E., et al. (2026). Identification of Parkinson's disease-associated regulatory variants in human dopaminergic neurons reveals modulators of SCARB2 and BAG3 expression. Preprint at bioRxiv. https://doi.org/10.64898/2026.03.26.714241.

Gérard, D., Schmidt, F., Ginolhac, A., Schmitz, M., Halder, R., Ebert, P., Schulz, M.H., Sauter, T., and Sinkkonen, L. (2019). Temporal enhancer profiling of parallel lineages identifies AHR and GLIS1 as regulators of mesenchymal multipotency. Nucleic Acids Res. *47*, 1141–1163. https://doi.org/10.1093/nar/gky1240.

Gomez Ramos, B., Ohnmacht, J., de Lange, N., Valceschini, E., Ginolhac, A., Catillon, M., Ferrante, D., Rakovic, A., Halder, R., Massart, F., et al. (2024). Multiomics analysis identifies novel facilitators of human dopaminergic neuron differentiation. EMBO Rep. *25*, 254–285. https://doi.org/10.1038/s44319-023-00024-2.

Ha, M., Kim, J., Park, S.M., Hong, C.M., Han, M.-E., Song, P., Kang, C.-D., Lee, D., Kim, Y.H., Hur, J., et al. (2020). Prognostic Role of Zinc Finger Homeobox 4 in Ovarian Serous Cystadenocarcinoma. Genet. Test. Mol. Biomark. *24*, 145–149. https://doi.org/10.1089/gtmb.2019.0185.

Hanss, Z., Larsen, S.B., Antony, P., Mencke, P., Massart, F., Jarazo, J., Schwamborn, J.C., Barbuti, P.A., Mellick, G.D., and Krüger, R. (2021). Mitochondrial and Clearance Impairment in p.D620N VPS35 Patient-Derived Neurons. Mov. Disord. *36*, 704–715. https://doi.org/10.1002/mds.28365.

Hao, Y., Stuart, T., Kowalski, M.H., Choudhary, S., Hoffman, P., Hartman, A., Srivastava, A., Molla, G., Madad, S., Fernandez-Granda, C., et al. (2024). Dictionary learning for integrative, multimodal and scalable single-cell analysis. Nat. Biotechnol. *42*, 293–304. https://doi.org/10.1038/s41587-023-01767-y.

Hegarty, S.V., Sullivan, A.M., and O'Keeffe, G.W. (2013). Midbrain dopaminergic neurons: A review of the molecular circuitry that regulates their development. Dev. Biol. *379*, 123–138. https://doi.org/10.1016/j.ydbio.2013.04.014.

Heinz, S., Benner, C., Spann, N., Bertolino, E., Lin, Y.C., Laslo, P., Cheng, J.X., Murre, C., Singh, H., and Glass, C.K. (2010). Simple Combinations of Lineage-Determining Transcription Factors Prime *cis*-Regulatory Elements Required for Macrophage and B Cell Identities. Mol. Cell *38*, 576–589. https://doi.org/10.1016/j.molcel.2010.05.004.

Hemmi, K., Ma, D., Miura, Y., Kawaguchi, M., Sasahara, M., Hashimoto-Tamaoki, T., Tamaoki, T., Sakata, N., and Tsuchiya, K. (2006). A homeodomain-zinc finger protein, ZFHX4, is expressed in neuronal differentiation manner and suppressed in muscle differentiation manner. Biol. Pharm. Bull. *29*, 1830–1835. https://doi.org/10.1248/bpb.29.1830.

Hnisz, D., Abraham, B.J., Lee, T.I., Lau, A., Saint-André, V., Sigova, A.A., Hoke, H.A., and Young, R.A. (2013). Super-enhancers in the control of cell identity and disease. Cell *155*, 934–947. https://doi.org/10.1016/j.cell.2013.09.053.

Jaroszewicz, A., and Ernst, J. (2023). Chromgene: gene-based modeling of epigenomic data. Genome Biol. *24*, 203. https://doi.org/10.1186/s13059-023-03041-5.

Jassal, B., Matthews, L., Viteri, G., Gong, C., Lorente, P., Fabregat, A., Sidiropoulos, K., Cook, J., Gillespie, M., Haw, R., et al. (2020). The reactome pathway knowledgebase. Nucleic Acids Res. *48*, D498–D503. https://doi.org/10.1093/nar/gkz1031.

Kamath, T., Abdulraouf, A., Burris, S.J., Langlieb, J., Gazestani, V., Nadaf, N.M., Balderrama, K., Vanderburg, C., and Macosko, E.Z. (2022). Single-cell genomic profiling of human dopamine neurons identifies a population that selectively degenerates in Parkinson's disease. Nat. Neurosci. *25*, 588–595. https://doi.org/10.1038/s41593-022-01061-1.

Kaya-Okur, H.S., Wu, S.J., Codomo, C.A., Pledger, E.S., Bryson, T.D., Henikoff, J.G., Ahmad, K., and Henikoff, S. (2019). CUT&Tag for efficient epigenomic profiling of small samples and single cells. Nat. Commun. *10*, 1930. https://doi.org/10.1038/s41467-019-09982-5.

Kent, L.N., and Leone, G. (2019). The broken cycle: E2F dysfunction in cancer. Nat. Rev. Cancer *19*, 326–338. https://doi.org/10.1038/s41568-019-0143-7.

Kim, J.-Y., Jeong, H.S., Chung, T., Kim, M., Lee, J.H., Jung, W.H., and Koo, J.S. (2017). The value of phosphohistone H3 as a proliferation marker for evaluating invasive breast cancers: A comparative study with Ki67. Oncotarget *8*, 65064–65076. https://doi.org/10.18632/oncotarget.17775.

Krämer, A., Green, J., Pollard, J., and Tugendreich, S. (2014). Causal analysis approaches in Ingenuity Pathway Analysis. Bioinformatics *30*, 523–530. https://doi.org/10.1093/bioinformatics/btt703.

Kramer, N.E., Davis, E.S., Wenger, C.D., Deoudes, E.M., Parker, S.M., Love, M.I., and Phanstiel, D.H. (2022). Plotgardener: cultivating precise multi-panel figures in R. Bioinformatics *38*, 2042–2045. https://doi.org/10.1093/bioinformatics/btac057.

Kriks, S., Shim, J.-W., Piao, J., Ganat, Y.M., Wakeman, D.R., Xie, Z., Carrillo-Reid, L., Auyeung, G., Antonacci, C., Buch, A., et al. (2011). Floor plate-derived dopamine neurons from hescs efficiently engraft in animal models of PD. Nature *480*, 547–551. https://doi.org/10.1038/nature10648.

La Manno, G., Gyllborg, D., Codeluppi, S., Nishimura, K., Salto, C., Zeisel, A., Borm, L.E., Stott, S.R.W., Toledo, E.M., Villaescusa, J.C., et al. (2016). Molecular Diversity of Midbrain Development in Mouse, Human, and Stem Cells. Cell *167*, 566–580.e19. https://doi.org/10.1016/j.cell.2016.09.027.

Li, H., Handsaker, B., Wysoker, A., Fennell, T., Ruan, J., Homer, N., Marth, G., Abecasis, G., and Durbin, R.; 1000 Genome Project Data Processing Subgroup (2009). The Sequence Alignment/Map format and samtools. Bioinformatics *25*, 2078–2079. https://doi.org/10.1093/bioinformatics/btp352.

Liao, Y., Smyth, G.K., and Shi, W. (2014). Featurecounts: an efficient general purpose program for assigning sequence reads to genomic features. Bioinformatics *30*, 923–930. https://doi.org/10.1093/bioinformatics/btt656.

Liao, Y., Smyth, G.K., and Shi, W. (2019). The R package Rsubread is easier, faster, cheaper and better for alignment and quantification of RNA sequencing reads. Nucleic Acids Res. *47*, e47. https://doi.org/10.1093/nar/gkz114.

Love, M.I., Huber, W., and Anders, S. (2014). Moderated estimation of fold change and dispersion for RNA-seq data with deseq2. Genome Biol. *15*, 550. https://doi.org/10.1186/s13059-014-0550-8.

Meyer-Lindenberg, A., Miletich, R.S., Kohn, P.D., Esposito, G., Carson, R.E., Quarantelli, M., Weinberger, D.R., and Berman, K.F. (2002). Reduced prefrontal activity predicts exaggerated striatal dopaminergic function in schizophrenia. Nat. Neurosci. *5*, 267–271. https://doi.org/10.1038/nn804.

Milacic, M., Beavers, D., Conley, P., Gong, C., Gillespie, M., Griss, J., Haw, R., Jassal, B., Matthews, L., May, B., et al. (2024). The Reactome Pathway Knowledgebase 2024. Nucleic Acids Res. *52*, D672–D678. https://doi.org/10.1093/nar/gkad1025.

Mölder, F., Jablonski, K.P., Letcher, B., Hall, M.B., Tomkins-Tinch, C.H., Sochat, V., Forster, J., Lee, S., Twardziok, S.O., Kanitz, A., et al. (2021). Sustainable data analysis with Snakemake. https://doi.org/10.12688/f1000research.29032.2.

Molinoff, P.B., and Axelrod, J. (1971). Biochemistry of catecholamines. Annu. Rev. Biochem. *40*, 465–500. https://doi.org/10.1146/annurev.bi.40.070171.002341.

Nakamura, E., Hata, K., Takahata, Y., Kurosaka, H., Abe, M., Abe, T., Kihara, M., Komori, T., Kobayashi, S., Murakami, T., et al. (2021). Zfhx4 regulates endochondral ossification as the transcriptional platform of Osterix in mice. Commun. Biol. *4*, 1258. https://doi.org/10.1038/s42003-021-02793-9.

Novak, G., Kyriakis, D., Grzyb, K., Bernini, M., Rodius, S., Dittmar, G., Finkbeiner, S., and Skupin, A. (2022). Single-cell transcriptomics of human ipsc differentiation dynamics reveal a core molecular network of Parkinson's disease. Commun. Biol. *5*, 49. https://doi.org/10.1038/s42003-021-02973-7.

Nowak, J.S., Choudhury, N.R., de Lima Alves, F., Rappsilber, J., and Michlewski, G. (2014). Lin28a regulates neuronal differentiation and controls mir-9 production. Nat. Commun. *5*, 3687. https://doi.org/10.1038/ncomms4687.

Ong, C.-T., and Corces, V.G. (2011). Enhancer function: new insights into the regulation of tissue-specific gene expression. Nat. Rev. Genet. *12*, 283–293. https://doi.org/10.1038/nrg2957.

Parker, S.C.J., Stitzel, M.L., Taylor, D.L., Orozco, J.M., Erdos, M.R., Akiyama, J.A., van Bueren, K.L., Chines, P.S., Narisu, N., Brian, L.B., et al. (2013). Chromatin stretch enhancer states drive cell-specific gene regulation and harbor human disease risk variants. Proc. Natl. Acad. Sci. USA *110*, 17921–17926. https://doi.org/10.1073/pnas.1317023110.

Pérez Baca, M.D.R., Palomares-Bralo, M., Vanhooydonck, M., Hamerlinck, L., D'haene, E., D'haene, E., Jacobs, E.Z., Cock, L.D., D'haenens, E., Malfait, Z., et al. (2025). Loss of function of the zinc finger homeobox 4 gene, *ZFHX4*, underlies a neurodevelopmental disorder. Am. J. Hum. Genet. *112*, 1388–1414. https://doi.org/10.1016/j.ajhg.2025.04.008.

Poulin, J.-F., Gaertner, Z., Moreno-Ramos, O.A., and Awatramani, R. (2020). Classification of Midbrain Dopamine Neurons Using Single-Cell Gene Expression Profiling Approaches. Trends Neurosci. *43*, 155–169. https://doi.org/10.1016/j.tins.2020.01.004.

Qing, T., Zhu, S., Suo, C., Zhang, L., Zheng, Y., and Shi, L. (2017). Somatic mutations in ZFHX4 gene are associated with poor overall survival of Chinese esophageal squamous cell carcinoma patients. Sci. Rep. *7*, 4951. https://doi.org/10.1038/s41598-017-04221-7.

R Core Team (2014). R: A Language and Environment for Statistical Computing (R Found. Stat. Comput).

Rakovic, A., Voß, D., Vulinovic, F., Meier, B., Hellberg, A.-K., Nau, C., Klein, C., and Leipold, E. (2022). Electrophysiological Properties of Induced Pluripotent Stem Cell-Derived Midbrain Dopaminergic Neurons Correlate With Expression of Tyrosine Hydroxylase. Front. Cell. Neurosci. *16*, 817198. https://doi.org/10.3389/fncel.2022.817198.

Reinhardt, P., Glatza, M., Hemmer, K., Tsytsyura, Y., Thiel, C.S., Höing, S., Moritz, S., Parga, J.A., Wagner, L., Bruder, J.M., et al. (2013). Derivation and expansion using only small molecules of human neural progenitors for neurodegenerative disease modeling. PLoS One *8*, e59252. https://doi.org/10.1371/journal.pone.0059252.

Ritchie, M.E., Phipson, B., Wu, D., Hu, Y., Law, C.W., Shi, W., and Smyth, G.K. (2015). Limma powers differential expression analyses for RNA-sequencing and microarray studies. Nucleic Acids Res. *43*, e47. https://doi.org/10.1093/nar/gkv007.

Sawamoto, N., Doi, D., Nakanishi, E., Sawamura, M., Kikuchi, T., Yamakado, H., Taruno, Y., Shima, A., Fushimi, Y., Okada, T., et al. (2025). Phase I/II trial of ips-cell-derived dopaminergic cells for Parkinson's disease. Nature *641*, 971–977. https://doi.org/10.1038/s41586-025-08700-0.

Schubert, M., Lindgreen, S., and Orlando, L. (2016). Adapterremoval v2: rapid adapter trimming, identification, and read merging. BMC Res. Notes *9*, 88. https://doi.org/10.1186/s13104-016-1900-2.

Sun, X., and Kaufman, P.D. (2018). Ki-67: more than a proliferation marker. Chromosoma *127*, 175–186. https://doi.org/10.1007/s00412-018-0659-8.

Tabar, V., Sarva, H., Lozano, A.M., Fasano, A., Kalia, S.K., Yu, K.K.H., Brennan, C., Ma, Y., Peng, S., Eidelberg, D., et al. (2025). Phase I trial of hes cell-derived dopaminergic neurons for Parkinson's disease. Nature *641*, 978–983. https://doi.org/10.1038/s41586-025-08845-y.

Thomaidou, D., Politis, P.K., and Matsas, R. (2010). Neurogenesis in the Central Nervous System: Cell Cycle Progression/Exit and Differentiation of Neuronal Progenitors. In Cell Cycle Regulation and Differentiation in Cardiovascular and Neural Systems, A. Giordano and U. Galderisi, eds. (New York, NY: Springer), pp. 141–175.

Tranchevent, L.-C., Halder, R., and Glaab, E. (2023). Systems level analysis of sex-dependent gene expression changes in Parkinson's disease. NPJ Park. Dis. *9*, 8. https://doi.org/10.1038/s41531-023-00446-8.

Tzschentke, T.M., and Schmidt, W.J. (2000). Functional Relationship Among Medial Prefrontal Cortex, Nucleus Accumbens, and Ventral Tegmental Area in Locomotion and Reward. Crit. Rev. Neurobiol. *14*, 131–142. https://doi.org/10.1615/critrevneurobiol.v14.i2.20.

Whyte, W.A., Orlando, D.A., Hnisz, D., Abraham, B.J., Lin, C.Y., Kagey, M.H., Rahl, P.B., Lee, T.I., and Young, R.A. (2013). Master transcription factors and mediator establish super-enhancers at key cell identity genes. Cell *153*, 307–319. https://doi.org/10.1016/j.cell.2013.03.035.

Wingett, S.W., and Andrews, S. (2018). Fastq Screen: A tool for multi-genome mapping and quality control. F1000Res. *7*, 1338. https://doi.org/10.12688/f1000research.15931.2.

Wolff, J., Bhardwaj, V., Nothjunge, S., Richard, G., Renschler, G., Gilsbach, R., Manke, T., Backofen, R., Ramírez, F., and Grüning, B.A. (2018). Galaxy hicexplorer: a web server for reproducible Hi-C data analysis, quality control and visualization. Nucleic Acids Res. *46*, W11–W16. https://doi.org/10.1093/nar/gky504.

Yu, G., Wang, L.-G., and He, Q.-Y. (2015). Chipseeker: an R/Bioconductor package for chip peak annotation, comparison and visualization. Bioinformatics *31*, 2382–2383. https://doi.org/10.1093/bioinformatics/btv145.

Yu, J., Vodyanik, M.A., Smuga-Otto, K., Antosiewicz-Bourget, J., Frane, J.L., Tian, S., Nie, J., Jonsdottir, G.A., Ruotti, V., Stewart, R., et al. (2007). Induced Pluripotent Stem Cell Lines Derived from Human Somatic Cells. Science *318*, 1917–1920. https://doi.org/10.1126/science.1151526.

Zetterström, R.H., Solomin, L., Jansson, L., Hoffer, B.J., Olson, L., and Perlmann, T. (1997). Dopamine neuron agenesis in Nurr1-deficient mice. Science *276*, 248–250. https://doi.org/10.1126/science.276.5310.248.

Zhang, Y., Liu, T., Meyer, C.A., Eeckhoute, J., Johnson, D.S., Bernstein, B.E., Nusbaum, C., Myers, R.M., Brown, M., Li, W., and Liu, X.S. (2008). Model-based Analysis of chip-Seq (MACS). Genome Biol. *9*, R137. https://doi.org/10.1186/gb-2008-9-9-r137.

Zhu, A., Ibrahim, J.G., and Love, M.I. (2019). Heavy-tailed prior distributions for sequence count data: removing the noise and preserving large differences. Bioinforma *35*, 2084–2092. https://doi.org/10.1093/bioinformatics/bty895.

Zong, S., Xu, P.P., Xu, Y.H., and Guo, Y. (2022). A bioinformatics analysis: ZFHX4 is associated with metastasis and poor survival in ovarian cancer. J. Ovarian Res. *15*, 90. https://doi.org/10.1186/s13048-022-01024-x.

## STAR★METHODS

### KEY RESOURCES TABLE

| REAGENT or RESOURCE | SOURCE | IDENTIFIER |
|---|---|---|
| **Antibodies** | | |
| Chicken polyclonal anti-MAP2 | Abcam | Cat# ab5392; RRID:AB_2138153 |
| Rabbit polyclonal anti-SOX2 | Abcam | Cat# ab97959; RRID:AB_2341193 |
| Mouse monoclonal anti-Ki67 | BD Pharmingen™ | Cat# 550609; RRID:AB_393778 |
| Rabbit polyclonal anti-phospho-Histone 3 (Ser10) | Sigma-Aldrich (Merck) | Cat# 06–570; RRID:AB_310177 |
| Mouse monoclonal anti-TH | Thermo Fisher Scientific | Cat# MA1-24654; RRID:AB_795666 |
| Rabbit polyclonal anti-ZFHX4 | Merck | Cat# HPA023837; RRID: AB_1234567 |
| Rabbit polyclonal anti-H3K27ac | Abcam | Cat# ab4729; RRID: AB_2118291 |
| Goat anti-chicken IgY (H + L), Alexa Fluor 568 | Invitrogen | Cat# A11041; RRID: AB_2534098 |
| Donkey anti-rabbit IgG (H + L), Alexa Fluor 568 | Invitrogen | Cat# A10042; RRID: AB_2534017 |
| Goat anti-chicken IgY (H + L), Alexa Fluor 647 | Invitrogen | Cat# A21449; RRID: AB_2535866 |
| Donkey anti-mouse IgG (H + L), Alexa Fluor 647 | Invitrogen | Cat# A31571; RRID: AB_162542 |
| Goat anti-rabbit IgG (H + L), Alexa Fluor 488 | Invitrogen | Cat# A11034; RRID: AB_2576217 |
| Goat anti-mouse IgG (H + L), Alexa Fluor 568 | Invitrogen | Cat# A11031; RRID: AB_144696 |
| **Bacterial and virus strains** | | |
| Lentivirus expressing shSscramble | This study | N/A |
| Lentivirus expressing shRNA targeting ZFHX4_b | This study | N/A |
| Lentivirus expressing shRNA targeting ZFHX4_c | This study | N/A |
| Lentivirus expressing shRNA targeting ZFHX4_b | This study | N/A |
| Lentivirus expressing shRNA targeting ZFHX4_c | This study | N/A |
| **Chemicals, peptides, and recombinant proteins** | | |
| DMEM/F-12 media | Gibco | Cat# 21331046 |
| Neurobasal medium | Gibco | Cat# 21103049 |
| N-2 Supplement (100X) | Gibco | Cat# 17502001 |
| B-27 Supplement (50X), minus vitamin A | Gibco | Cat# 12587010 |
| Penicillin-Streptomycin (10,000 u/mL) | Gibco | Cat# 15140122 |
| GlutaMAX Supplement | Gibco | Cat# 35050061 |

(*Continued on next page*)

*Continued*

| REAGENT or RESOURCE | SOURCE | IDENTIFIER |
|---|---|---|
| Purmorphamine | Sigma-Aldrich | Cat# SML0868 |
| L-Ascorbic acid | Sigma-Aldrich | Cat# A4544 |
| CHIR99021 | Axon Medchem | Cat# AXON1386 |
| Fibroblast Growth Factor 8b (FGF8b) | PeproTech | Cat# 10025 |
| Geltrex | Gibco | Cat# A1413302 |
| Dibutyryl cAMP (dbcAMP) | Santa Cruz Biotechnology | Cat# sc-201567C |
| Brain-Derived Neurotrophic Factor (BDNF) | PeproTech | Cat# 450-02 |
| Glial Cell Line-Derived Neurotrophic Factor (GDNF) | PeproTech | Cat# 450-10 |
| Transforming Growth Factor β3 (TGF-β3) | PeproTech | Cat# 100-36E |
| Ampicillin | Sigma-Aldrich | Cat# A9518 |
| LB broth | Sigma-Aldrich | Cat# L7658 |
| Fetal Bovine Serum (FBS) | Thermo Fisher Scientific | Cat# 26400044 |
| Calcium chloride ($CaCl_2$) | Sigma-Aldrich | Cat# 21115 |
| HEPES-buffered saline (HBS) | Sigma-Aldrich | Cat# 51558 |
| Chloroquine | Sigma-Aldrich | Cat# C6628 |
| Accutase | Sigma-Aldrich | Cat# A6964 |
| Phosphate-Buffered Saline (PBS) | Gibco | Cat# 14190185 |
| dNTP mix | Thermo Fisher Scientific | Cat# R0181 |
| RiboLock RNase Inhibitor | Thermo Fisher Scientific | Cat# E00381 |
| RevertAid Reverse Transcriptase | Thermo Fisher Scientific | Cat# EP0442 |
| Oligo(dT)$_{18}$ primer | Eurogentec | Cat# UN-PR100-005 |
| Absolute Blue qPCR SYBR Green Low ROX Mix | Thermo Fisher Scientific | Cat# AB4322B |
| TaqMan Fast Advanced Master Mix | Thermo Fisher Scientific | Cat# 4444557 |
| Hoechst 33342 | Invitrogen | Cat# H21492 |
| Paraformaldehyde | Sigma-Aldrich | CAS: 30525-89-4 |
| Phosphate-buffered saline (PBS), with calcium and magnesium | Gibco | Cat# 14040-091 |
| Triton X-100 | Sigma-Aldrich | Cat# X100 |
| Normal Goat Serum | Vector Laboratories | Cat# S-1000 |
| Bovine Serum Albumin (BSA), Fraction V, IgG-free | Carl Roth | Cat# 3737.2 |
| DAPI (4′,6-diamidino-2-phenylindole dihydrochloride) | Sigma-Aldrich | CAS: 28718-90-3 |
| Accumax Cell Aggregate Dissociation Medium | Thermo Fisher Scientific | Cat# 00-4666-56 |
| Formaldehyde | Sigma-Aldrich | Cat# 1.03999 |

(*Continued on next page*)

*Continued*

| REAGENT or RESOURCE | SOURCE | IDENTIFIER |
|---|---|---|
| Glycine | Carl Roth | Cat# 3908.3 |
| Sodium dodecyl sulfate (SDS) | Carl Roth | Cat# 2326.1 |
| MboI restriction enzyme | New England Biolabs | Cat# R0147L |
| DNA Polymerase I, Large (Klenow) Fragment | New England Biolabs | Cat# M0210L |
| T4 DNA Ligase Buffer | Thermo Fisher Scientific | Cat# EL0011 |
| Phenol:Chloroform:Isoamyl alcohol (25:24:1) | Sigma-Aldrich | Cat# 77617 |
| Ethanol | Carl Roth | Cat# 9065.2 |
| RNase A | Thermo Fisher Scientific | Cat# EN0531 |
| T4 DNA Polymerase | New England Biolabs | Cat# M0203S |
| Biotin-14-dCTP | Thermo Fisher Scientific | Cat# 19518018 |
| Dynabeads MyOne Streptavidin C1 | Thermo Fisher Scientific (Invitrogen) | Cat# 65001 |
| SPRIselect Beads | Beckman Coulter | Cat# B23318 |
| **Critical commercial assays** | | |
| CUT&Tag-IT Express Assay Kit, Anti-Rabbit | Active Motif | Cat# 53160 |
| TaqMan MicroRNA Assay (hsa-miR-9) | Thermo Fisher Scientific | Assay ID: 000583 |
| TaqMan MicroRNA Assay (U6 snRNA) | Thermo Fisher Scientific | Assay ID: 001973 |
| TaqMan MicroRNA Reverse Transcription Kit | Thermo Fisher Scientific | Cat# 4366596 |
| Quick-RNA Microprep Kit | Zymo Research | Cat# R1050 |
| NucleoBond Xtra Midi EF kit | Macherey-Nagel | Cat# 740420.50 |
| **Deposited data** | | |
| RNA-seq | Gomez Ramos et al. (2024) | EGA: EGAD00001009288 |
| ATAC-seq | Gomez Ramos et al. (2024) | EGA: EGAD00001009288 |
| ChIP-seq data | Gomez Ramos et al. (2024) | EGA: EGAD00001009288 |
| RNA-seq | This study | EGA: EGAD50000001605 |
| CUT&Tag data | This study | EGA: EGAD50000001605 |
| LowC data | Gérard et al. (2026), available on biorxiv | EGA: EGAC00001002822 |
| PD brain transcriptomics (RNA-seq) | Tranchevent et al. (2023) | GEO: GSE8397 |
| Human substantia nigra snRNA-seq | Kamath et al. (2022) | GEO: GSE178265; Broad SCP: SCP1768, SCP1769 |
| Raw sequencing data (snRNA-seq) | Kamath et al. (2022) | Dbgap: phs002879.v1.p1 |
| Parkinson's Progression Markers Initiative (PPMI) dataset | PPMI database | RRID: SCR_006431; https://www.ppmi-info.org |
| **Experimental models: Cell lines** | | |
| HEK 293T cells | ATCC | Cat# CRL-3216; RRID: CVCL_0063 |
| TH-Rep1 reporter cell line (CRISPR/Cas9-engineered from GM17602 iPSC) | Gomez Ramos et al. (2024) | N/A |

(*Continued on next page*)

***Continued***

| REAGENT or RESOURCE | SOURCE | IDENTIFIER |
|---|---|---|
| GM17602 iPSC line (control) | Coriell Institute for Medical Research | Cat# GM17602 |
| One Shot Stbl3 Chemically Competent *E. Coli* | Thermo Fisher Scientific | Cat# C7373-03 |
| **Oligonucleotides** | | |
| (see Table S7) | – | – |
| **Software and algorithms** | | |
| R | R Core Team (2014) | Version 4.3.0, 4.3.2; https://www.r-project.org/ |
| GraphPad Prism | SCR_002798 | Version 11.0.0; RRID: SCR_002798; https://www.graphpad.com |
| FlowJo | SCR_008520 | Version 10.10.1; RRID: SCR_008520; https://www.flowjo.com |
| ImageJ | SCR_003070 | Version 1.54g; RRID: SCR_003070; https://imagej.nih.gov/ij/ |
| HOMER | Heinz et al. (2010) | http://homer.ucsd.edu/homer |
| Seurat | Hao et al. (2024) | Version 4.1.1; https://satijalab.org/seurat |
| Limma | Ritchie et al. (2015) | https://bioconductor.org/packages/limma |
| DESeq2 | Love et al. (2014) | https://bioconductor.org/packages/deseq2 |
| Rsubread | Liao et al. (2019) | https://bioconductor.org/packages/Rsubread |
| Snakemake | Mölder et al. (2021) | https://snakemake.readthedocs.io |
| STAR | Dobin et al. (2013) | https://github.com/alexdobin/STAR |
| SAMtools | Li et al. (2009) | http://www.htslib.org |
| FastQ Screen | Wingett and Andrews (2018) | https://www.bioinformatics.babraham.ac.uk/projects/fastq_screen |
| AdapterRemoval | Schubert et al. (2016) | https://adapterremoval.readthedocs.io |
| MACS3 | Zhang et al. (2008) | https://github.com/macs3-project/MACS |
| featureCounts | Liao et al. (2014) | Https://subread.sourceforge.net |
| snakemake-cut-n-tag workflow | This study | Version 0.1.0; Docker: ginolhac/snake-cut-and-tag:0.3 |
| MATLAB (R2024a Update 6) | MathWorks | Version 24.1.0.2689473 |
| HiCExplorer | Wolff et al. (2018) | Version 3.7.6; https://hicexplorer.readthedocs.io |
| plotgardener | Kramer et al. (2022) | Version 1.16.0; https://bioconductor.org/packages/plotgardener |
| **Other** | | |
| 96-well flat-bottom plate | Greiner Bio-One | Cat# 655090 |
| PhenoPlate 96-well plate | PerkinElmer | Cat# 6055308 |

## EXPERIMENTAL MODEL AND STUDY PARTICIPANT DETAILS

### Cell lines

The TH-Rep1 reporter cell line was derived from the human induced pluripotent stem cell (iPSC) line GM17602 (Coriell), which also served as a control during fluorescence-activated cell sorting (FACS) in this study. This iPSC line has been utilized and described in previous studies (Gomez Ramos et al., 2024; Rakovic et al., 2022), where it was used to establish a reporter system. To generate the TH-Rep1 line, a CRISPR/Cas9-based approach was employed to introduce a biallelic insertion of a T2A-mCherry sequence at the stop codon of the endogenous tyrosine hydroxylase (TH) gene. Routine testing confirmed that the cell lines remained free of mycoplasma contamination.

HEK293T (ATCC, CRL-3216) were cultured in DMEM/F12 (Gibco) supplemented with 10% fetal bovine serum (FBS, ThermoFisher Scientific, 26400044) and 1% penicillin-streptomycin (Gibco, Cat# 15140122) and maintained at 37°C in a humified incubator with 5% $CO_2$.

### Ethics statement

Ethical approval for experiments with human iPSC-derived cell types was obtained from the Ethics Review Panel of University of Luxembourg (Reference ERP 25-031 EpiCNS).

## METHOD DETAILS

### Cell culture and differentiation

The protocol for differentiating small molecule neural progenitor cells (smNPCs) into mDANs was adapted from a previously published method (Hanss et al., 2021). Cells were plated onto Geltrex-coated culture dishes. smNPCs were cultured in N2B27 medium composed of a 1:1 mixture of DMEM/F12 and Neurobasal medium, supplemented with N-2 Supplement (100X; Gibco, Cat# 17502001), B27 Supplement (50X) (minus vitamin A) (Gibco, Cat# 12587010), 1% Penicillin-Streptomycin, GlutaMax Supplement. Cells were maintained at 37°C in a humified incubator with 5% $CO_2$. For maintenance of smNPCs, the N2B27 medium was additionally supplemented with 500 nM Purmorphamine (PMA) (Sigma-Aldrich, SML0868), 150 μM L-ascorbic acid (AA) (Sigma-Aldrich, A4544) and 3 μM CHIR99021 (CHIR) (Axon Medchem, AXON1386). During the initial differentiation phase (day0-8), cells were treated with N2B27 medium supplemented with 100 ng/ml FGF8b (PeproTech, Cat# 100-25), 1 μM PMA, and 200 μM ascorbic acid. From day 8 to day 10, cells were cultured in N2B27 containing 0.5 μM of PMA and 200 μM L-ascorbic acid. From day 10 onwards, cells were maintained in maturation medium composed of N2B27 containing 200 μM ascorbic acid, along with 10 ng/ml brain-derived neurotrophic factor (BDNF; PeproTech, Cat# 450-02), 10 ng/ml glial cell line-derived neurotrophic factor (GDNF; PeproTech, Cat# 450-10), 500 μM dibutyryl cAMP (dbcAMP; Santa Cruz Biotechnology, Cat# sc-201567C), and 1 ng/ml transforming growth factor β3 (TGF-β3; PeproTech, Cat# 100-36E).

### Bacterial culture, plasmid extraction and lentivirus production

ZFHX4 knockdown was achieved using a third-generation lentiviral delivery system. For viral particle production, HEK293T cells were co-transfected with the requisite packaging plasmids (pMDG, pMDL, and pREV) and the lentiviral expression vector called psi-LVRU6GP, encoding either an shRNA directed against ZFHX4 (HSH112197-LVRU6GP-b, HSH112197-LVRU6GP-c) or a scrambled control (CSHCTR001-LVRU6GP). In this expression vector, shRNA expression is driven by the U6 promoter, while the SV40 promoter regulates the GFP reporter, enabling assessment of transduction efficiency through GFP expression.

Glycerol bacteria stocks containing the plasmid of interest were amplified in 5 ml LB Broth medium (Sigma-Aldrich) supplemented with 100 μg/ml ampicillin (Sigma-Aldrich, A9518-5G). At 37°C with shaking at 120 rpm for 5 hours, then inoculated into an Erlenmeyer flask containing 150 mL of LB Broth with ampicillin, followed by an overnight incubation under the same conditions. After bacterial expansion, plasmid extraction was performed using the NucleoBond Xtra Midi EF kit (Macherey-Nagel), according to the manufacturer's protocol. For lentiviral production, $8 \times 10^6$ HEK293T cells were seeded into a T75 flask with 15 ml of DMEM/F12 (Gibco) supplemented with 10% fetal bovine serum (FBS, ThermoFisher Scientific, 26400044) and 1% penicillin-streptomycin and maintained at 37°C in a humified incubator with 5% $CO_2$. The following day, cells were transfected using the third-generation lentiviral system. A DNA mixture consisting of 4 μg pMDG, 2 μg pMDL, 2 μg pREV, and 8 μg of the transfer plasmid was prepared in 200 μl of 1 M $CaCl_2$ (Sigma), and the volume was adjusted to 800 μl with sterile water. This solution was gently combined with

800 μl of HEPES-buffered saline (Sigma-Aldrich, 51558-50ML) and incubated at room temperature for 20 minutes. Meanwhile, 16 μl of 25 mM chloroquine (Sigma-Aldrich, C6628) was added to the HEK293T cells and incubated for at least 5 minutes. The prepared transfection complex was then added to the cells. After 4–6 hours, the medium was replaced with 14 ml of fresh DMEM/F12. Viral supernatant was collected after 48 hours, centrifuged at 2000 rpm for 10 minutes at 4°C, and filtered through a 0.45 μm membrane (Sartorius). The resulting viral particles were aliquoted into 1 ml portions and stored at −80°C.

### Transduction

Lentiviral transduction of differentiating neurons was carried out at specific time points during the protocol, as outlined in Figure 2A. Cells were pre-seeded at $1 \times 10^6$ on day 0 for early transduction and $3 \times 10^6$ on day 8 for late transduction. On the day of transduction, frozen lentiviral particles were thawed on ice, and fresh differentiation medium was prepared. The medium was removed from each well, and the required volume of virus was added. For ZFHX4 KD, the transduction was performed using a 1:1 mixture of lentiviral particles encoding shZFHX4-b and shZFHX4-c. If the viral volume was less than 1 ml, it was supplemented with medium to reach this amount. Plates were sealed and centrifuged at 300 × g for 10 minutes at room temperature to enhance transduction efficiency. Subsequently, additional medium was added to each well to reach a final volume of 2 ml. Media were refreshed in less than 24 hours post-transduction. All lentiviral vectors included a GFP marker to monitor transduction efficiency and viral preparations were titrated beforehand to achieve approximately 80% efficiency, confirmed via GFP expression by flow cytometry. Knockdown efficiency was validated by quantitative RT–PCR and ICC, as shown in the results section.

### Flow cytometry and FACS

Cells were dissociated using 800 μl of Accumax (Thermo Fisher Scientific) and incubated at 37°C for approximately 5–10 minutes. Two volumes of DMEM/F-12 were added and the cell suspension was filtered through CellTrics® strainers (50 μm, CAT no. 04-004-2325, Sysmex) into a 15 ml falcon tube. After centrifugation at 300 × g for 3 minutes at room temperature (RT), the pellet was gently resuspended in PBS and transferred to 1.5 ml tubes. Hoechst 33342 (Invitrogen™-H21492) was added to a final concentration of 1 μg/ml, and the samples were rotated at 4°C for 5 minutes. For dead cell control, one sample was heat-inactivated at 70°C for 5 minutes. The cells were then washed by centrifugation under the same conditions and resuspended in PBS for further analysis. Flow cytometry was performed using the BD LSRFortessa™ analyzer while for FACS, BD FACSMelody™ Cell Sorter was used for purifying cells, finally data were analyzed using FlowJo (version 10.10.1). The gating strategy is shown in Figure S5.

### Total RNA extraction, cDNA synthesis and RT-qPCR

Total RNA extraction was performed by first aspirating the medium and adding 600 μl of Lysis Buffer from the Quick-RNA Microprep Kit (Zymo Research) directly to each well. RNA isolation was carried out according to the manufacturer's guidelines. RNA concentration was determined using a NanoDrop spectrophotometer. For cDNA synthesis, between 300 ng and 1 μg of RNA, depending on sample availability, was diluted in nuclease-free water to reach a total volume of 27 μl. 13 μl of master mix was then added, containing 1× cDNA synthesis buffer, 0.5 mM dNTPs (ThermoFisher Scientific, R0181), 2.5 μM oligo(dT) primer, 1 U/μl Ribolock RNase inhibitor (ThermoFisher Scientific, EO0381), and 5 U/μl RevertAid Reverse Transcriptase (ThermoFisher Scientific, EP0442), bringing the final reaction volume to 40 μl. Samples were incubated at 42°C for 1 hour followed by 70°C for 10 minutes to terminate the reaction. The cDNA was diluted with nuclease-free water, with dilution factors of 1:10 for 1 μg RNA or 1:3 for 300 ng RNA. RT-qPCR reactions were set up in a total volume of 20 μl, containing 5 μl of cDNA and 15 μl of master mix prepared with 1×Absolute Blue SYBR Green ROX Low Mix (thermofisher Scientific, 283 AB4322B), 500 nM of each primer, and nuclease-free water. Reactions were run in triplicate on MicroAmp™ Fast Optical 96-Well Reaction Plate using an Applied Biosystems 7500 Fast Real-Time PCR System or the QuantStudio 12K Flex Real Time PCR System under the cycling conditions of 95°C for 15 minutes, followed by 40 cycles of 95°C for 15 seconds, 55°C for 15 seconds, and 72°C for 30 seconds. Gene expression analysis was performed using the 2ˆ−(ΔΔct) method, where ΔΔct was calculated as: (Δct_target − Δct_housekeeping) − (Δct_target − Δct_housekeeping) reference condition. ACTB was used as the reference gene, and expression levels were normalized to the reference condition. Statistical significance was evaluated using either one-sample t-test or ANOVA.

### TaqMan assay

To evaluate miR-9 expression, the TaqMan™ MicroRNA Assay specific for hsa-miR-9 (ThermoFisher, Assay ID: 000583) was employed together with the U6 snRNA (Assay ID: 001973) as an internal reference. Reverse transcription was

performed with the TaqMan™ MicroRNA Reverse Transcription Kit (Thermo Fisher Scientific, Cat# 4366596), following the supplier's instructions. 3 μl of 5× RT primer was mixed with 5 μl of RNA template containing 1–10 ng of nucleic acid. The mixture was initially incubated at 85°C for 5 minutes and then at 60°C for 5 minutes. Subsequently, 7 μl of a reaction mixture was added, comprising 0.15 μl of 100 mM dNTPs (with dTTP), 1 μl of MultiScribe™ Reverse Transcriptase (50 U/μl), 1.5 μl of 10× RT Buffer, 0.19 μl of RNase inhibitor (20 U/μl), and 4.16 μl of nuclease-free water. The reverse transcription was performed under the following conditions: 30 minutes at 16°C, followed by 30 minutes at 42°C, and a final step at 85°C for 5 minutes. For RT-PCR, 1.33 μl of the synthesized cDNA was combined with 1 μl of 20× TaqMan™ Small RNA Assay, 10 μl of TaqMan™ Fast Advanced Master Mix, and 7.67 μl of nuclease-free water to a final volume of 20 μl. Amplification was carried out on the Applied Biosystems 7500 Fast Real-Time PCR System. The cycling protocol followed indicated 50°C for 2 minutes, 95°C for 20 seconds, then 40 cycles of 95°C for 3 seconds and 60°C for 30 seconds. Relative quantification was performed using the $2^{-\Delta\Delta Ct}$ method as previously described.

### CRISPR activation

CRISPR activation (CRISPRa) was used to overexpress ZFHX4. The plasmid pLV hU6-gRNA-hUbC VP64-dCas9-VP64-T2A-GFP (Addgene #66707) was employed for lentiviral production and constitutive expression of dCas9-VP64 after transduction. The selected gRNAs targeting ZFHX4 and a scramble control were cloned into the backbone of this plasmid using Golden Gate assembly with the BsmBI type IIS restriction enzyme. The cloning of the gRNA sequences was performed by GeneCust. Following plasmid delivery, One Shot™ Stbl3™ Chemically Competent E. Coli (Thermo Fisher Scientific, Cat# C7373-03) were transformed following the manufacturer's instructions. Glycerol stocks were prepared, and plasmid isolation and lentivirus production were performed as previously described.

### Live-cell imaging-based proliferation assay

At day 0, $1 \times 10^6$ cells per condition (shZFHX4 and shSCRAMBLE) were seeded into Geltrex-coated 6-well plates. Early transduction was performed on day 1 as described in the dedicated section for shSCRAMBLE and shZFHX4. On day 2, the medium was replaced and the plates were transferred to the Omni Pro 12™ live-cell imaging system. Brightfield images were acquired every 12 hours from day 2 to day 8 using a 10× objective. For each well, nine non-overlapping fields were imaged. Confluence (%) was automatically quantified using the instrument's integrated image analysis software based on segmentation of the cell-covered area relative to the total image area. The mean confluence per well was calculated from the nine fields. The culture medium was refreshed every two days.

### Immunocytochemistry

Cells were seeded into a PhenoPlate 96-well (PerkinElmer, 6055308) or 96-well flat-bottom plate (Greiner Bio-One, cat. No. 655090), according to the assay. On the day of analysis, cells were fixed with 50 μl of 4% paraformaldehyde for 15 minutes at RT. After fixation, cells were washed three times with PBS containing $MgCl_2$ and $CaCl_2$ (Gibco). Permeabilization was performed by incubating the cells for 15 minutes with 75 μl of PBS containing $MgCl_2$, $CaCl_2$, 0.4% Triton X-100, 10% normal goat serum, and 2% BSA. The cells were then washed twice with PBS containing $MgCl_2$ and $CaCl_2$, followed by overnight incubation at 4°C with 50 μl of primary antibody diluted appropriately in PBS with $MgCl_2$, $CaCl_2$, 0.1% Triton X-100, 1% goat serum, and 0.2% BSA. After three washes with PBS containing $MgCl_2$ and $CaCl_2$, cells were incubated for 1 hour at RT with 50 μl of secondary antibody diluted in the same buffer as the primary antibody. Following three additional washes, cells were incubated for 15 minutes at RT with DAPI (1 μg/mL) and washed again twice. Fixed and stained cells were stored at 4°C until analysis.

### High-content imaging-based time-course analysis of mDAN differentiation

All cells were seeded in the same 96-well flat-bottom plate (Greiner Bio-One, cat. No. 655090). Cells collected on day 8, day 15 and smNPCs originated from independent differentiation experiments and were plated at different time points to ensure synchronized collection. Day 8 cells were seeded on day 0 at 80,000 cells per well, while day 15 cells were seeded on day 7 at the same density. smNPCs were seeded at 20,000 cells per well two days prior collection. All samples were fixed on the day of collection. The primary antibodies used were rabbit polyclonal anti-ZFHX4 1:100 (Merck-HPA023837), mouse monoclonal anti TH 1:400 (Thermo Fisher Scientific, MA1-24654), rabbit polyclonal anti-SOX2 1:250 (ab97959) with their respective secondary antibodies diluted 1:1000, goat anti-Rabbit Alexa Fluor™ 488 (Invitrogen—A11034) and goat anti-mouse Alexa Fluor™ 568 (Invitrogen—A11031). For each well, sixteen images were acquired at 20x magnification using the CellVoyager CV8000 High Content Screening System (Yokogawa). DAPI fluorescence was excited with a 405 nm laser and collected through a 445/45 nm bandpass filter. Alexa Fluor 488 was excited using a

488 nm h a 525/50 nm bandpass filter. Alexa Fluor 568 was excited using a 561 nm laser and emission was detected through a 600/37 nm bandpass filter. Alexa Fluor 647 was excited with a 640 nm laser, and emission was collected using a 676/29 nm bandpass filter. Signal masks for ZFHX4, SOX2, and TH were generated using a custom MATLAB pipeline based on intensity thresholding. For each marker, signal area and intensity above threshold were quantified and normalized to DAPI. Single-cell co-expression analysis was performed using nucleus-based segmentation, with nuclei identified by DAPI staining and assigned unique Cell IDs. TH and ZFHX4 signals were quantified within each nuclear mask by counting positive pixels. Cells were classified as positive if $\geq 1$ corresponding fluorescent pixel was detected within the nucleus. The fraction of $TH^+$ cells co-expressing ZFHX4 was calculated as the number of double-positive cells divided by the total number of $TH^+$ cells.

### High-content imaging proliferation assay following ZFHX4 KD

Cells were seeded into a PhenoPlate 96-well (PerkinElmer, 6055308) at 100 000 densities at day 4, while at day 8 of analysis cells were fixed and stained as prior described. The primary antibodies used were rabbit polyclonal anti-ZFHX4 1:100 (Merck-HPA023837), mouse monoclonal anti-Ki67 1:100 (BDPharmingen 550609), rabbit polyclonal anti-PH3 1:500 (Merck-06-570), chicken polyclonal anti-MAP2 1:1000 (Abcam-ab5392) with their respective secondary antibodies diluted 1:1000 goat anti-Chicken Alexa Fluor™ 568 (Invitrogen—A11041), donkey anti-Rabbit Alexa Fluor™ 568 (Invitrogen— A10042), goat anti-Chicken Alexa Fluor™ 647(Invitrogen—A21449) and donkey anti-Mouse Alexa Fluor™ 647 (Invitrogen— A31571). For each well, sixteen image z-stacks were acquired at 20x magnification using the CellVoyager CV8000 High-Content Screening System (Yokogawa). Each z-stack consisted of three focal planes spaced 3.2 μm apart. DAPI fluorescence was excited with a 405 nm laser and collected through a 445/45 nm bandpass filter. Alexa568 was excited using a 561 nm laser and emission was detected through a 600/37 nm bandpass filter. Alexa647 excitation was performed with a 640 nm laser, with emission captured behind a 676/29 nm bandpass filter. Signal masks for ZFHX4, Ki67, PH3 and MAP2 were created using a pipeline developed in Matlab, by applying thresholding. The signal intensity of each marker, in regions exceeding the threshold, was then calculated and normalized over DAPI.

### Sequencing

RNA quality was assessed with the Agilent RNA 6000 Nano kit on an Agilent 2100 Bioanalyzer. Only samples with a RIN greater than 7 were selected for sequencing. Three independent biological replicates per condition, scrambled control and ZFHX4 knockdown, were sequenced. RNA sequencing was performed using the Illumina stranded mRNA library preparation kit, generating paired-end reads of 50 base pairs on a NovaSeq6000 platform. The quality of the CUT&Tag libraries was defined using the Agilent High Sensitivity DNA Kit on the Agilent 2100 Bioanalyzer. The sequencing was carried out in a Nextseq2000 machine using paired-end 75 bp read length.

### RNA-seq data analysis

For the RNA-seq data from ZFHX4 knock down experiments a snakemake pipeline was used (Mölder et al., 2021). This pipeline includes the tools STAR, SAMtools, FastQC, FastQ Screen, AdapterRemoval, Rsubread, DESeq2, ggplot2 and apeglm (Dobin et al., 2013; Li et al., 2009; Liao et al., 2019; Love et al., 2014; Schubert et al., 2016; Wingett and Andrews, 2018; Zhu et al., 2019). For more details about the pipeline, please refer to our repository (RNA-seq folder, RNA-seq_DataAnalysis_TF_KDs.rmd script). The genome version and annotation were GRCh38 release 102. The analysis pipeline was based on the software container https://hub.docker.com/layers/ginolhac/snake-rna-seq/0.4.

### Identification of SE-controlled transcription factors

Integrative analysis of chromatin accessibility and transcriptional profiles was performed using the previously published EPIC-DREM pipeline, adapted for ATAC-seq (Gérard et al., 2019; Gomez Ramos et al., 2024). Footprinting analyses based on chromatin accessibility enabled the identification of TF binding sites, which were then integrated with time-series transcriptional changes to construct gene regulatory networks underlying mDAN differentiation. These analyses yielded lists of TFs that control most of the observed transcriptional dynamics at each time point. Low-input H3K27ac ChIP-seq was carried out at days 30 and 50 to identify transcription factors regulated by super-enhancers. Enhancers and SEs were defined using HOMER (Heinz et al., 2010), with SEs classified as regulatory regions exceeding 10 kb in length. The genomic coordinates of TFs selectively expressed at each time point were overlapped with SE regions, resulting in a list of 49 SE-controlled TFs across the two time-points (Table S5). TFs differentially expressed at each stage of mDAN differentiation (day 15, day 30 and day 50) were further filtered for those showing a log2-fold change > 1 compared with smNPCs. Differentially expressed TFs were also filtered for those upregulated relative to non-mDANs on day 15 of

differentiation. The four resulting lists of upregulated TFs were then overlapped to identify factors consistently upregulated across conditions. This analysis yielded 33 TFs with selective expression in mDANs (Table S6). Finally, the list of 33 TFs was intersected with the list of 49 SE-controlled TFs, identifying 7 TFs that are both under SE control and selectively expressed in mDANs.

### Low chromosome conformation capture (LowC)

LowC data were generated for a separate study (Gérard et al., 2026). The workflow was performed on 110,000 sorted TH-Rep1 smNPCs or 30-day differentiated mDANs as described in detail in the original publication. Shortly, the cells were cross-linked with formaldehyde, quenched with glycine, washed, and lysed to isolate nuclei. Chromatin was permeabilized with SDS, quenched with Triton X-100, and digested using MBoI restriction enzyme, followed by enzyme inactivation. DNA overhangs were filled in with biotinylated nucleotides using Klenow polymerase, and proximity ligation was carried out with T4 DNA ligase to join spatially adjacent DNA fragments. After reverse cross-linking and protein digestion, DNA was purified by phenol-chloroform extraction and ethanol precipitation, treated with RNase A, and biotin was removed from unligated fragments using T4 DNA polymerase. The DNA was then sonicated, and biotinylated fragments were captured with streptavidin coated magnetic beads. End repair, adaptor ligation, USER treatment, and PCR amplification were performed to generate sequencing libraries, followed by SPRI bead-based size selection and quality control using a high-sensitivity DNA assay.

### LowC data analysis

LowC sequencing files produced by (Gérard et al., 2026) were processed using the Juicer pipeline as described here: https://github.com/sysbiolux/PD-rsnp_SCARB2/tree/master/Juicer_pipeline. The merged replicates were then converted to cool files using HiCExplorer (v3.7.6). The merged replicates were then converted to cool files using HiCExplorer (v3.7.6) (Wolff et al., 2018). smNPC and mDAN30 condition were scaled to the smallest condition using a resolution of 25 kb. Triangle representation of those chromatin contacts were then plotted using the R package plotgardener (v1.16.0) (Kramer et al., 2022) in R statistical software (v4.2.3) (R Core Team, 2014). The number of sequencing unique mapped reads obtained per sample was as follows: smNPC_01 (386,895,501), smNPC_02 (382,902,549), smNPC_03 (266,461,290), mDAN_D30_01 (257,293,787), mDAN_D30_02 (106,455,789), and mDAN_D30_03 (340,711,141).

### CUT&Tag

Three independent biological replicates were generated for smNPCs (for both H3K27ac and ZFHX4) and three independent biological replicates of sorted mDANs were produced for each condition. At day of analysis, mDANs were detached using 800 µl of Accumax (Thermo Fisher Scientific) for 5-10 minutes at 37°C. Two volumes of DMEM/F-12 were added, the cell suspension was filtered through CellTrics® strainers (50 µm, CAT no. 04-004-2325, Sysmex) into a 15 ml falcon tube and centrifuged at 300 × g for 3 minutes at RT. The pellet was then resuspended in PBS and transferred to 1.5 ml tubes. DAPI (1 µg/ml) was added to the samples and incubated for 5 minutes on a rotator at 4°C. After centrifugation with the same conditions, cells were washed twice in PBS containing 2% BSA and were then ready for sorting. For fluorescence-activated cell sorting (FACS), the BD FACSAria™ III sorter was used. 250 000 cells were obtained for each condition, after sorting. At day of analysis, smNPCs were detached using 750 µl of Accutase (Thermo Fisher Scientific) for 3 minutes at 37°C and 250 000 cells were collected per sample. Subsequent steps were performed identically for smNPCs and mDANs. Cells were passed to 1.5 ml tubes and centrifuged for 3 min, 600g at RT, the pellet was then resuspended in PBS and 0.6 ul of formaldehyde 19% were added. Samples were incubated for 2 minutes at RT and 7.5 µl of 1M glycine were added to the cell suspension to stop the cross-linking reaction. CUT&Tag was then performed using the CUT&Tag-IT® Express Assay Kit, Anti-Rabbit, 53160, from Active Motif as per manufacturer's instructions. The antibodies employed were the rabbit polyclonal anti-ZFHX4 (Merck-HPA023837) and the rabbit polyclonal anti-Histone H3 acetyl K27 (ab4729). 1 µg of each antibody was added to 50 µL of solution, resulting in a final concentration of 0.02 µg/ µL.

### CUT&Tag data analysis

The CUT&Tag analysis of H3K27ac and ZFHX4 in smNPC and mDANs on day 15 was performed using the snakemake-cut-n-tag workflow (v0.1.0) within the Docker container ginolhac/snake-cut-and-tag:0.3. Raw reads were quality-controlled, adapter-trimmed, and aligned to the human reference genome (GRCh38, Ensembl release 104). Duplicates were removed and reads with MAPQ < 30 were discarded. Peaks were called for each replicate using MACS3 (q < 0.01) and merged across samples, retaining regions detected in at least three replicates (Zhang et al., 2008).

Read counts per merged peak were obtained with featureCounts and normalized using scaling factors computed with ChIPseqSikeInFree (Liao et al., 2014). Differential enrichment between conditions was assessed using DESeq2, incorporating scaling factors into size factor estimation. Scaled coverage tracks (bigWig) and heatmaps were generated for visualization.

### Transcriptomic analysis of lateral SNc in PD

Gene expression data from a PD case-control brain transcriptomics study (Tranchevent et al., 2023), was downloaded from the Gene Expression Omnibus (GEO; dataset GSE8397), focusing on lateral substantia nigra samples from PD patients and healthy controls. Differential gene expression analysis was performed on the log-scaled data using the limma package in R (Ritchie et al., 2015). A linear model was fitted than adjusted for age and gender as covariates when comparing PD cases to controls.

### Analysis of single-nuclei RNA-seq of human SNc

Briefly, data (Kamath et al., 2022) were retrieved from the visualization tool download section (https://singlecell.broadinstitute.org/single_cell/study/SCP1768/, April 2025) for the following seven main cell classes: DA neurons, non DA neurons, OPC, Astro, Olig, Endo, and MG. Data included count matrices, metadata (cell type), and dimensional reduction as UMAP coordinates, estimated separately for each cell type. We selected data from cells present in metadata to build a Seurat (version 4.1.1) (Hao et al., 2024) object in R (version 4.3.0)(R Core Team, 2014). Counts were additionally log-normalised using Seurat's NormalizeData function and the object saved for downstream analyses and visualization of gene expression.

### Oligonucleotides

The oligonucleotides used in the study are listed in Table S7.

## QUANTIFICATION AND STATISTICAL ANALYSIS

Statistical analyses were performed using Graphpad Prism (version 11.0.0) and R (Version 4.3.2). Unpaired t-test or one-way ANOVA were used for statistical tests, as indicated in the corresponding figure legends. Data are represented as mean ±1 standard deviation (SD), unless otherwise indicated. The exact sample size (N) and its definition are specified in the corresponding figure legends. Statistical significance was defined as follows: * = p-value < 0.05, ** = p-value < 0.01, *** = p-value < 0.001, **** = p-value < 0.0001, and ns = not significant.

Stem Cell Reports, Volume *21*

## Supplemental Information

# ZFHX4 is necessary for dopaminergic neuron differentiation and controls cell cycle by regulating LIN28A

**Elena Valceschini, Borja Gomez Ramos, Jochen Ohnmacht, Aurelien Ginolhac, Marie Catillon, Deborah Gerard, Anthoula Gaigneaux, Dimitrios Kyriakis, Kamil Grzyb, Enrico Glaab, Anne Grünewald, Alexander Skupin, Thomas Sauter, Rejko Krüger, and Lasse Sinkkonen**

## Figure S1

A

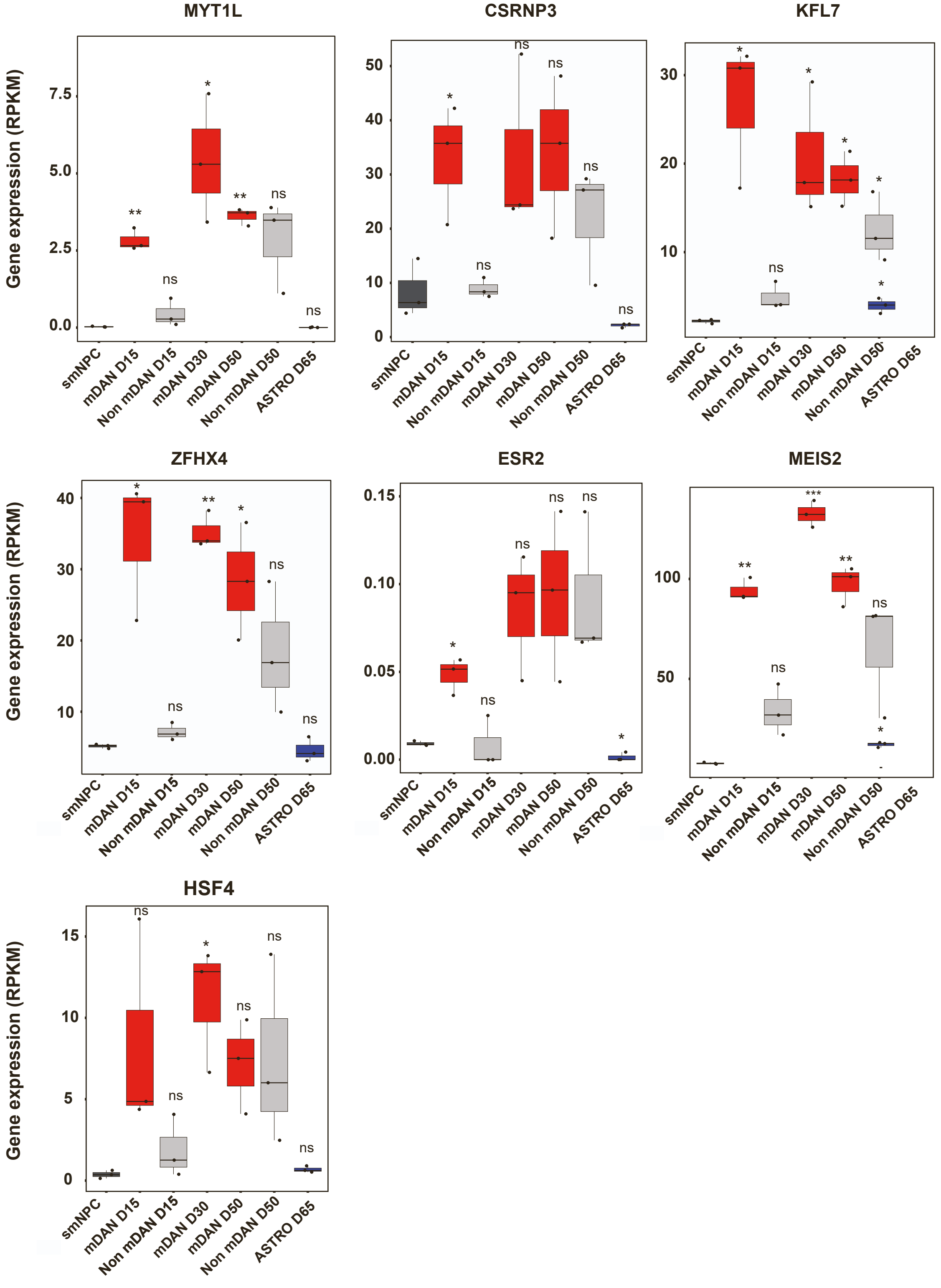


**Figure S1: Expression of 7 TFs across mDAN differentiation.** A) Expression dynamics of the 7 candidate TFs during mDAN differentiation of the TH-Rep1 cell line.

**Figure S2**

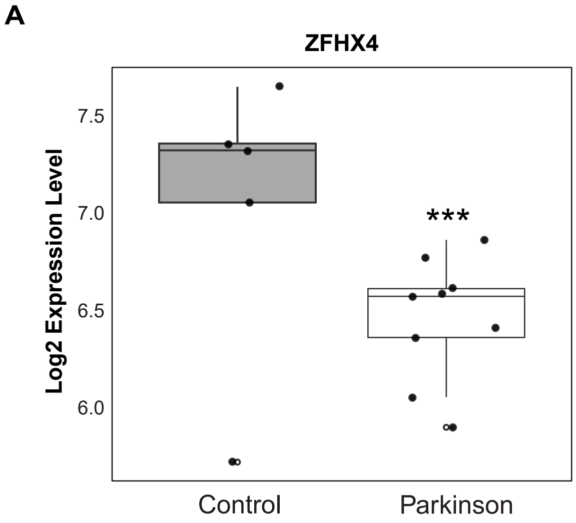


**Figure S2: ZFHX4 expression is reduced in PD.** A) Box plot showing ZFHX4 expression levels in lateral substantia nigra tissue from PD patients and healthy controls. Individual data points are overlaid on box plots, showing median, interquartile range, and outliers.

## Figure S3

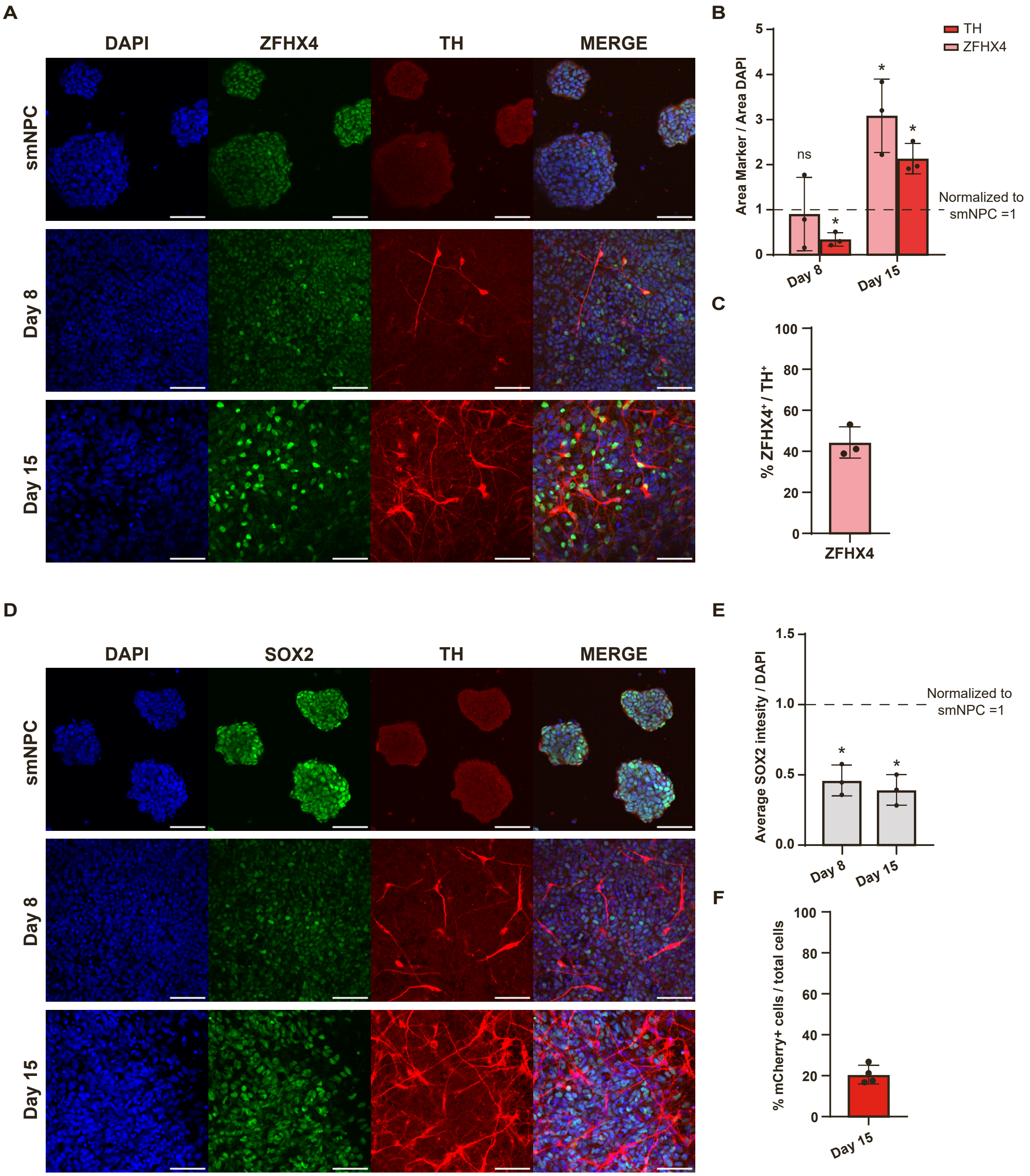


**Figure S3: mDAN maturation requires the induction of ZFHX4.** A) Representative images of the nuclear marker DAPI, ZFHX4, TH and the merged signal, in smNPC, day 8 of differentiation and day 15. B) Quantification of ZFHX4 and TH Area over Area of DAPI. Ratios were normalized to smNPC within each replicate (N = 3 independent experiments). C) Proportion of cells that are both TH positive and ZFHX4 positive in terms of intensity signal (N = 3 independent experiments). D) Representative images of the nuclear marker DAPI, SOX2, TH and the merged signal in smNPC, day 8 of differentiation and day 15. E) Quantification of SOX2 and TH Area over Area of DAPI. Ratios were normalized to smNPC per replicate (N = 3 independent experiments). F) Flow cytometry-based quantification of mCherry+ neurons at day 15 in TH-Rep1 reporter cell line compared with the unreporter control cell line (N = 4 independent experiments). Error bars (B, C, E, F), correspond to ±1 standard deviation (SD) from the mean. t-test (B, E), * = p-value < 0.05, ** = p-value < 0.01, *** = p-value < 0.001, **** = p-value < 0.0001, and ns = not significant.

## Figure S4

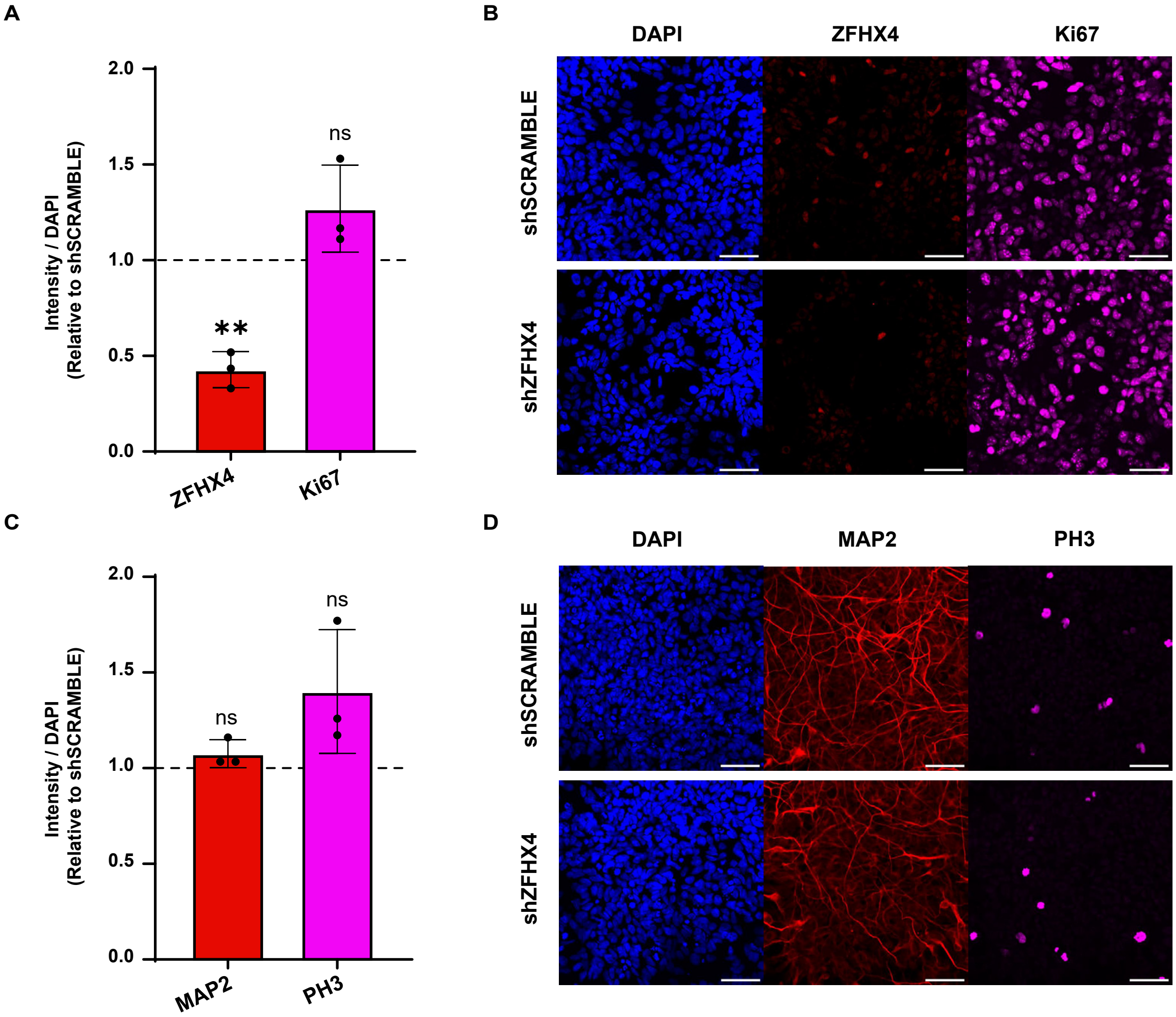


**Figure S4: Proliferation markers tend to increase upon ZFHX4 depletion.** A) Quantification of Ki67 or ZFHX4 positive-area normalized to DAPI-stained area. Ratios were normalized to shSCRAMBLE within each replicate. Analysis was performed on day 8 following early ZFHX4 KD (N = 3 independent experiments). (B) Representative images showing the nuclear marker DAPI, ZFHX4 and the proliferation marker Ki67. C) Quantification of MAP2 or PH3 positive-area normalized to DAPI stained area. Ratios were normalized to shSCRAMBLE within each replicate. Analysis was performed on day 8 following early ZFHX4 KD (N = 3 independent experiments). (D) Representative images of the nuclear marker DAPI, the neuronal marker MAP2 and the mitosis marker PH3. Error bars correspond to ±1 standard deviation (SD) from the mean, t-test, * = p-value < 0.05, ** = p-value < 0.01, *** = p-value < 0.001, **** = p-value < 0.0001, and ns = not significant.

## Figure S5

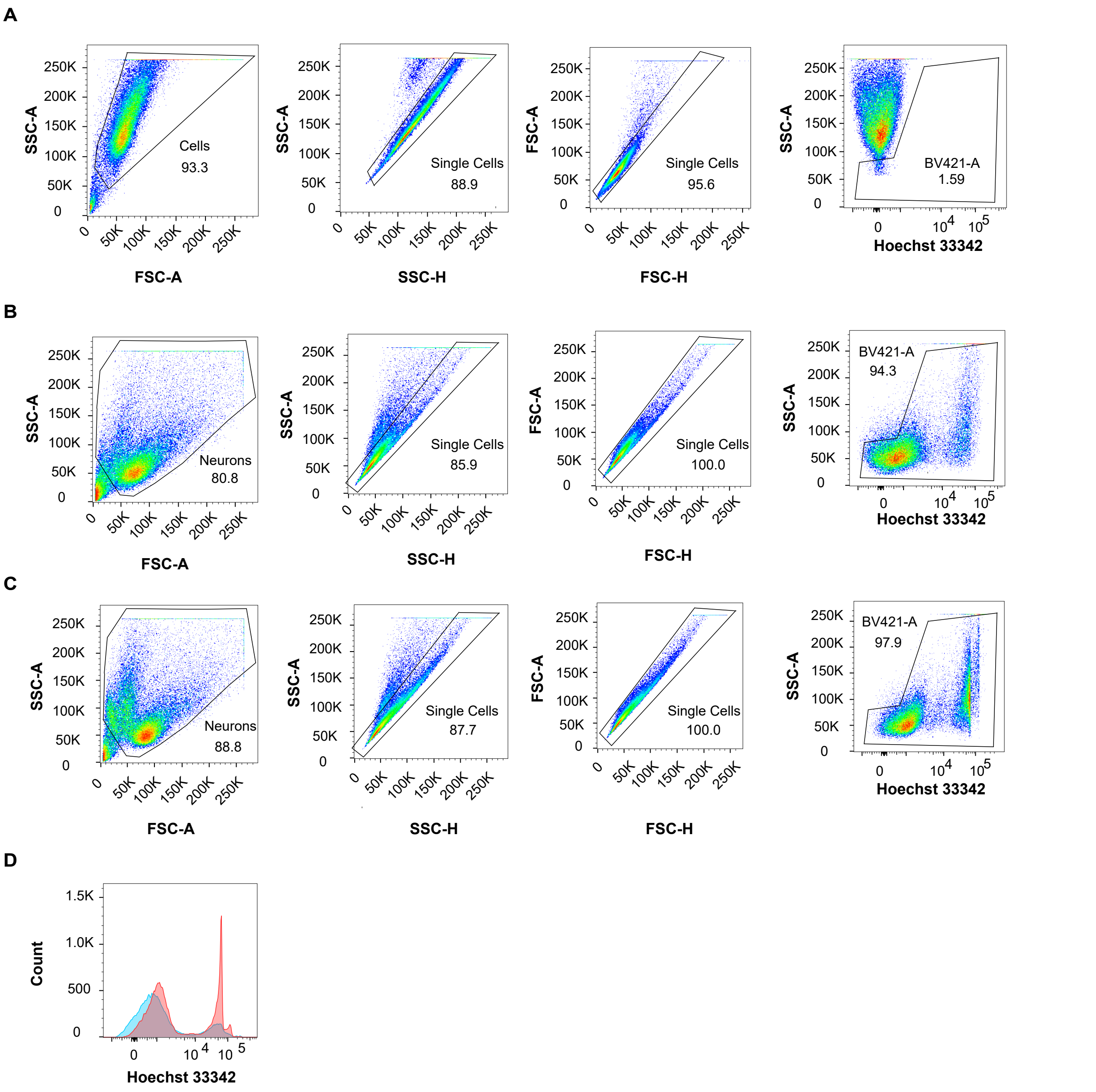


**Figure S5: Gating strategy for the cell cycle analysis upon ZFHX4 KD.** A) Representative gating of a dead mDAN control culture used to define the non-viable population. B) Gating strategy for the shSCRAMBLE condition. C) Gating strategy for the shZFHX4 condition. All samples were stained under the same conditions.
